# Which behaviour change techniques work best for diabetes self-management mobile apps? Results from a systematic review and meta-analysis of randomised controlled trials

**DOI:** 10.1016/j.ebiom.2024.105091

**Published:** 2024-04-05

**Authors:** Rosanna Tarricone, Francesco Petracca, Liv Svae, Maria Cucciniello, Oriana Ciani

**Affiliations:** a Department of Social and Political Sciences, Bocconi University, Milan, Italy; b Centre for Research on Health and Social Care Management (CERGAS), Government, Health and Not for Profit Division, SDA Bocconi School of Management, Milan, Italy

**Keywords:** Behaviour change, Behavioural theories, Self-management, Digital technologies, Mobile apps, Diabetes

## Abstract

**Background:**

Self-management is pivotal in addressing noncommunicable diseases, such as diabetes. The increased availability of digital behaviour change interventions (DBCIs) delivered through mobile health apps offers unprecedented opportunities to enhance self-management and improve health outcomes. However, little is known about the characteristics of DBCIs for diabetes that significantly impact glycaemic control. Therefore, our systematic review with meta-analysis aimed to summarize characteristics and behaviour change components in DBCIs for diabetes self-management and explore potential associations with metabolic outcomes.

**Methods:**

A systematic search was conducted in PubMed, Embase, the Cochrane Central Register of Controlled Trials, and Scopus to identify randomized controlled trials published until November 2023. The main outcome variable was the change in the mean difference of HbA1c levels between baseline and follow-up across intervention and control groups. Random-effects meta-regression was used to explore variation in glycaemic control as a function of prespecified characteristics of study designs and app interventions.

**Findings:**

A total of 57 studies was included in the analysis, showing a statistically significant percentage point reduction in HbA1c for the intervention group compared to the control arm (−0.36, 95% CI = −0.46 to −0.26, *p* < 0.001). The inclusion of “self-monitoring of behaviour” as a behaviour change technique (*β* = −0.22, *p* = 0.04) and “taking medication” as a target behaviour (*β* = −0.20, *p* = 0.05) was associated with improved metabolic outcomes.

**Interpretation:**

Our analyses endorse the use of diabetes self-management apps, highlighting characteristics statistically associated with intervention effectiveness and guiding the design of more effective DBCIs.

**Funding:**

This project received funding from the 10.13039/501100007601European Union’s Horizon 2020 programme.

**Research in context**

**Evidence before this study**

Effective self-management is essential in handling noncommunicable diseases like diabetes, yet its implementation encounters significant challenges. Advances in digital technologies provide new opportunities to modify health behaviours and enhance self-management. While experimental evidence consistently shows that mobile apps contribute to improved glycaemic control, little is known about the temporal contours of this relationship, the specific components of app interventions associated with improved health outcomes, and their connection with behaviour change theories. To contribute to this discussion, we conducted a search on PubMed, Embase, the Cochrane Central Register of Controlled Trials, and Scopus for randomized controlled trials published until November 2023 that employed app-based interventions to support self-management of diabetes and assessed their impact on glycaemic control.

**Added value of this study**

Drawing from the 57 studies included in this systematic review, we confirmed that mobile apps effectively support diabetes self-management and improve glycaemic control. The effectiveness of app-based DBCIs remained consistent across all time points, with progressive improvement up to nine months into the intervention, after which a decline in effectiveness was observed. Few studies explicitly referred to a theoretical basis in their intervention design. Additionally, our meta-regression verified that improvements in glycaemic control were associated with “self-monitoring of behaviour” as a behaviour change technique and “taking medication” as a target behaviour.

**Implications of all the available evidence**

Offering insights into characteristics of app-based DBCIs positively associated with effect size, this study can contribute to the design of more effective app-based interventions for diabetes self-management.

Nevertheless, significant challenges impede our empirical understanding of what works best to support self-management and behaviour change when applying DBCIs to high-prevalence chronic conditions like diabetes. Advancing behavioural theories tailored for the specificities of DBCIs and adopting innovative study designs for higher-quality evidence are essential steps. Additionally, understanding how the impact on clinical outcomes is mediated by intermediate effects on behavioural outcomes such as physical activity and medication adherence levels is equally crucial.

## Introduction

Self-management, defined as the “individual's ability to manage the symptoms, treatment, physical and psychosocial consequences and lifestyle changes inherent in living with a chronic disease”,^1^ is an essential component in the management of noncommunicable diseases (NCDs), emphasizing the individual responsibility to avoid preventable unhealthy behaviours and deal with medical and emotional management.^2^^,^^3^ Developing a set of skilled behaviours is particularly vital for diabetes, a disorder of the endocrine system largely driven by socio-economic and environmental factors. This condition poses significant public health concerns, affecting approximately 537 million adults between 20 and 79 years of age, with projections exceeding 1.31 billion by 2050 worldwide.^4^ Diabetes-related mortality rates, coupled with a global economic burden estimated at US $1.31 trillion and 1.8% of global gross domestic product (GDP) in 2015 and projected to substantially rise to $2.2 trillion (2.2% of global GDP) by 2030,^5^ further emphasize the need for effective self-management.

Diabetes self-management education and support (DSMES) provides the foundation to help all people with diabetes manage their self-care, leading to better physical well-being and enhanced quality of life, ultimately improving outcomes in glycaemic control, measured by the haemoglobin A1c (HbA1c) value.^6^^,^^7^ Despite evidence supporting the impact of DSMES across diabetes types and age groups,^8^, ^9^, ^10^, ^11^ sustained enactment of effective diabetes self-management in practice remains challenging. First, it entails actively engaging in diverse behavioural activities throughout the lifelong course of the illness.^12^ This holds true for both type 1 (insulin-dependent) and type 2 (non-insulin-dependent) diabetes, as they share comparable behavioural challenges and necessitate similar self-care behaviours.^13^ These include consistently adapting diet, maintaining a regular exercise schedule, monitoring evolving health status, and following complex medication regimens.^14^ Furthermore, diabetes management is largely independent of direct medical oversight, requiring patients to rely on their own resources to withstand the disease burden,^15^ and not easy to standardize, varying on a day-to-day basis in response to blood glucose volatility.^16^

Recently, advances in digital technologies have created unparalleled opportunities to constantly assess and modify health behaviours. Digital behaviour change interventions (DBCIs), utilising technologies such as computer programs, mobile apps and wearable devices to encourage behaviour change,^17^ offer novel technology-driven opportunities to facilitate self-management,^18^ and support patients with diabetes in addressing the daily difficulties they encounter.^13^^,^^18^^,^^19^

Notably, diabetes DBCIs are the most common among those targeting NCDs.^20^ The digital diabetes care market was projected to be worth US $1.5 billion in 2024,^21^ with top-funded companies offering DBCIs for type two diabetes already securing over US $2.4 billion in funding as of June 2021.^22^ In Germany, the first country where health apps assessment is linked with statutory coverage and reimbursement policies, five diabetes apps are listed as prescribable in the DIGA Directory at the end of the first quarter of 2024.^23^^,^^24^

Among DBCIs, apps benefit from instantaneous accessibility and promise to enhance intervention effectiveness through personalisation, tailored responses to real-time individual inputs, and just-in-time adaptation.^25^ With the continuous reinforcement of strategies to tackle traditional challenges like participant engagement and retention,^26^ apps thereby provide unprecedented opportunities for self-management by delivering complex interventions based on behaviour change techniques (BCTs), “observable, replicable, and irreducible components of an intervention designed to alter or redirect causal processes that regulate behaviour with minimum delivery specifications”.^27^ Identifying suitable BCTs should be guided by theories proposing models of human and health behaviour,^28^ as interventions based on a theoretical framework are generally considered more effective in changing behaviour than non-theory-based interventions.^29^ Several theories that either identify mediators of behaviour change or provide a philosophical foundation for underlying interventions have been shown to be associated with improved diabetes management and outcomes.^16^^,^^30^ As traditional programs encounter issues related to timing and limited flexibility to adapt to patients’ preferences and commitments,^31^ in-person interventions have gradually transitioned to digital delivery, and new digitally-native interventions are emerging.

Systematic reviews of randomized trials consistently support the effectiveness of app-based DBCIs in improving glycaemic control.^32^, ^33^, ^34^, ^35^, ^36^, ^37^, ^38^, ^39^ Despite favourable findings, these studies seldom explored the underlying theoretical basis of the interventions,^40^ with behavioural theory often labelled as the missing ingredient in digital tools in diabetes,^41^ and further evaluations necessary to ascertain which components best induce positive behaviour change.^42^ Previous studies have made preliminarily contributions in this area. Greenwood et al. (2017) proposed the technology-enabled self-management (TES) feedback loop, outlining key elements of technology-enabled interventions associated with significant changes in HbA1c.^43^ Similarly, other studies assessed brief digital behaviour change solutions for patients with type 2 diabetes^44^ or examined the relationship between intervention effect sizes of DBCIs and their features, including the number and type of BCTs tapped.^45^^,^^46^

Despite the extensive scope of prior analyses, given the lightning pace of technological development and the subsequent surge in published studies, more recent evidence is necessary to broaden our understanding of areas that remain unexplored. This systematic review thus aims to summarize the intervention characteristics, BCTs and behavioural theories included in diabetes self-management apps tested in experimental studies published until November 2023, discuss the temporal dynamics of the relationship between app use and glycaemic control, and explore associations between specific features or modes of delivery characteristics and metabolic outcomes.

## Methods

This systematic review with meta-analysis was conducted and reported in accordance with the Preferred Reporting Items for Systematic Reviews and Meta-Analyses (PRISMA) statement (Supplementary Data 1).^47^ The review was not pre-registered, and a protocol was not prepared. This study was deemed exempt from institutional ethical approval.

### Eligibility criteria

Studies with randomized designs recruiting individuals with a diagnosis of either type 1 or type 2 diabetes were included, with no additional restriction criteria based on age, sex, ethnicity or comorbidities. Interventions included patient-facing mobile apps targeting diabetes self-management through behaviour change strategies encompassing at least one BCT. Apps were intended as self-contained software only accessible through mobile technologies such as smartphones and tablets. Valid comparators could be usual care or a less intensive digital intervention not containing the active ingredient of the main intervention, such as telephone follow-up, SMS messaging, or a digital placebo. When multiple intervention arms including the use of an app were present, we chose the one that allowed us to isolate the direct contribution of the app alone, minimizing potential confounding effects due to other non-digital components of the intervention (such as additional human-led activities). In terms of outcomes, included studies had to measure the impact of the app on glycaemic control measured through HbA1c levels, either as a primary or secondary outcome, expressed in % or in mmol/mol. In terms of report characteristics, we exclusively focused on peer-reviewed studies published in English starting from 2008, when app stores were initially released. The exclusion criteria comprised: i) women with gestational diabetes or individuals at risk of developing the disease, including those diagnosed with pre-diabetes); ii) interventions exclusively relying on SMS technology or where the mobile app only played a subsidiary role in a complex, multi-faceted intervention; iii) DBCIs provided to healthcare professionals (or caregivers) only; iv) interventions with insufficient details in the main text, and in associated publications, to map the included BCTs.

### Information sources

We searched PubMed, Embase, the Cochrane Central Register of Controlled Trials in the Cochrane Library, and Scopus to identify relevant studies published until November 2023. The search was initially conducted on 17 April 2022 and later updated on 3 May and 11 December 2023. Additional records were identified by looking at the reference lists of studies eligible for full-text review and systematic reviews retrieved by the initial search. The search strategy was iteratively refined and adapted to each specific database. A detailed illustration of the sequence of terms employed across all databases is presented in Supplementary Data 2.

### Selection and data collection process

Two reviewers (FP, LS) independently screened titles and abstracts of all records retrieved using the selected search strategies. Potentially eligible articles were analysed full-text independently by the same reviewers based on the inclusion and exclusion criteria. At the full-text analysis stage, the main reason for exclusion was recorded for ineligible studies. Data from selected records was collected by LS and checked by FP, based on a prespecified data extraction form. At every stage of this process, any inconsistencies were initially discussed between the two reviewers and reconciled together with a third researcher (RT), if necessary.

### Data items

The outcome variable for this analysis was the change in the mean difference of HbA1c levels between baseline and follow-up across intervention and control groups expressed in percentage points. Effect measures were computed from mean changes and standard deviations (SD) for both the intervention and the control arm at all available time points. Whenever data was not explicitly reported in the text, authors were contacted and asked to provide the missing information. When we did not receive a reply from the authors and graphic-only results were available, data points were estimated through the Web Plot Digitizer software. If SD was missing but authors reported standard error (SE) or confidence intervals (CI), we estimated SD using the formula outlined in the Cochrane Handbook.^48^ The same source was used for imputing within-group SDs for changes from baseline, using a correlation coefficient calculated as the average of the correlation coefficients from studies reported in considerable detail and including baseline, post-intervention and change SDs. If SDs for changes from baseline were available for one time point only, it was hypothesized equal for the other time points as well. When median and interquartile ranges only were reported, we adopted the method proposed by Wan et al. (2014), as detailed in Supplementary Data 3.^49^ In addition to effect size information, data on the following variables was extracted from the retrieved records: (a) study information; (b) characteristics of participants; (c) intervention period; (d) mode of intervention delivery; (e) app characteristics (features of the development process, self-care behaviours targeted, content of the intervention, level of technology automation, additional professional involvement). Study information included data on target population in terms of subtype of diabetes, inclusion criteria, recruitment mode and sample sizes. For studies with more than two arms, the number of participants included in the analysis refers to the sum of participants in the selected intervention and control groups only. Participant characteristics included data on average age of participants, proportion of males and females in the sample, and baseline HbA1c levels. The intervention period was categorized as 3, 6, 9 or 12 months, approximating to the closest of these time points if the measurement timeframe was not precisely aligned. The intervention could be delivered either through a study device provided to all study participants at baseline or by adopting a bring-your-own-device (BYOD) approach and consequently downloading the app on the participant's smartphone. Information on the development features of the apps (i.e., strategies and considerations adopted throughout the development stage) were retrieved in terms of: i) grounding on any behavioural theories as reported by the authors; and ii) user and/or clinician involvement throughout the design stages, including for usability testing. The intervention was also classified in terms of self-care behaviours targeted by the app to promote successful and effective diabetes self-management by adopting the ADCES7 Self-Care Behaviours (ADCES7) framework.^50^ To characterize the active ingredients of the interventions, we adopted the BCT taxonomy v1 (BCTTv1) developed by Michie et al. (2013), which includes 93 techniques and 16 higher-order groupings and has been widely adopted in behavioural science.^51^ Two researchers (FP and LS) independently coded the content of the interventions based on what authors reported in the main text and in retrievable, associated publications (such as study protocols and trial records). Only BCTs uniquely detected in experimental arms were coded as “included” in the analysis, strategically isolating the active ingredients to test and allowing for more profound insights into the intervention–outcome relationship analysis.^52^ Both reviewers completed the BCTTv1 online training course. Technology automation was classified through a dummy variable as either present or absent, based on whether the app autonomously supported decision making or provided direct recommendations to patients in the light of the collected or inputted data. Similarly, additional healthcare professional involvement was used to code any incremental human-led support offered by a professional to participants in the intervention arm, either through the app or via other offline channels.

### Risk of bias assessment

Risk of bias in the included studies was assessed in accordance with the Revised Cochrane risk-of-bias tool (RoB 2) using HbA1c as the outcome under consideration.^53^ The pertinent version of the tool was used to assess the quality in individual-randomized and cluster RCTs. RoB 2 is structured into five specific bias domains: (i) randomization process; (ii) deviations from intended interventions; (iii) missing outcome data; (iv) measurement of the outcome; (v) selection of the reported result. Judgements were defined for each domain, and a synthetic summary of the overall risk of bias was subsequently categorized as either “low risk”, “some concerns”, or “high risk”. Given the anticipated concerns related to the blinding process and potential deviations from interventions, studies flagged with concerns in only one domain were assessed as having a low overall risk of bias, while studies at high risk of bias for at least one domain or judged to have “some concerns” in three or more domains were marked as having a high risk of bias. One researcher (FP) performed the quality assessment of studies, and a second (OC) independently double-checked the assessment, with disagreements resolved by consensus. Figures were plotted using the Robvis tool, a web application aiding in the visualization of risk of bias.^54^

### Data synthesis and analysis

Kappa statistics were calculated to evaluate the level of agreement between the two reviewers during the study selection process and the BCT coding. Given the assumption that different studies are estimating different, yet related effects, and the projected differences in intervention contents and study characteristics, we adopted a random-effects meta-analysis model using a restricted maximum likelihood variance estimator to assess the average distribution of the amount by which the experimental intervention changes the outcome compared with the control. In the main model, all studies were included using the effect size associated with the latest time-point. Additional models were run at each time point (3, 6, 9 and 12 months from randomization) including all the studies reporting effect measures at that time point with the aim of modelling the temporal relationship between app interventions and health outcomes. One final model included only the studies reporting effect sizes at both the 3- and 6-month time point to analyse the trend of the impact of app interventions over time in a subset of comparable studies. Statistical heterogeneity in the meta-analysis was assessed through the Chi-square test and the *I*^*2*^ statistic to describe the proportion of total variability due to between-study heterogeneity (48). In case of moderate (*I*^*2*^ ≥50%) and high (*I*^*2*^ ≥75%) statistical heterogeneity, we planned to carry out subgroup analyses to inspect the causes of heterogeneity and assess the robustness of the synthesized results by grouping included studies by overall risk of bias, identification of HbA1c levels as primary or secondary outcome, and study design (distinguishing pilot and full-scale RCTs). Furthermore, we used random-effects meta-regression with restricted maximum likelihood estimation to explore variation in glycaemic control as a function of prespecified characteristics of study designs and app interventions. Meta-regression is a statistical technique capable of identifying predictors of effect size from characteristics of individual trials.^55^ Study-level trial features and participant characteristics, together with the presence or absence of each BCT, the total number of techniques tapped and the ADCES7 self-care behaviours targeted by the app were initially defined as independent variables for random-effects univariate meta-regression models. The association between each of the covariates and intervention effectiveness was assessed when the presence of each covariate was detected in at least 10% and no more than 90% of the included studies, a criterion similarly used in previous meta-regressions for BCTs.^56^^,^^57^ A multivariate random-effects meta-regression model was then planned by including as covariates all study characteristics and BCTs that demonstrated a meaningful association with effect size (*p* < 0.05) in the univariate model. In our analysis, the resulting regression coefficients (*β*) are the estimated impact in the effect measure if that specific covariate was included in the app intervention: negative, statistically significant (*p* < 0.05) coefficients hence indicate that interventions had a greater improvement in glycaemic control than the comparators when that covariate was included. Adjusted *R*^*2*^ was analysed to assess how much of the outcome heterogeneity was accounted for by the covariate(s) included in each model.

A series of sensitivity analyses was run to assess the robustness of the overall effect estimate by sequentially removing each study and analysing the impact on the direction and extent of the association. A contour-enhanced funnel plot was used to visually inspect the risk of bias due to missing results and assess small-study effects. If asymmetry in the funnel plot was detected, we planned to assess whether the asymmetry was likely attributable to publication bias through the Egger test and trim-and-fill method using the rightmost-run estimator.^58^ All analyses were conducted using STATA SE 18.0 (StataCorp, College Station, TX).

### Role of the funding source

The funder was not involved in the study design, the collection, analysis and interpretation of data, the writing of the report, and the decision to submit the paper for publication.

## Results

**Fig. 1:**
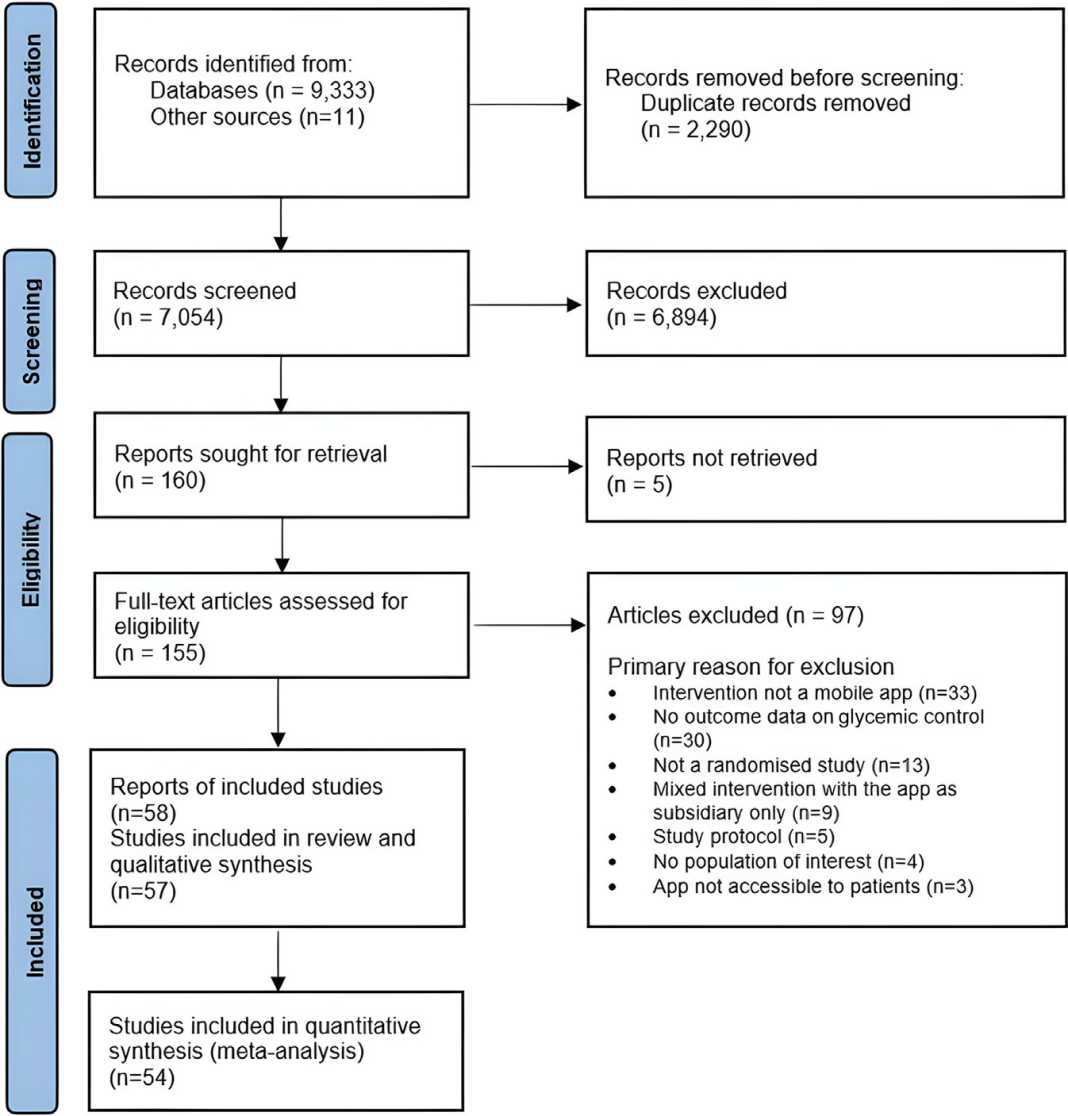
PRISMA flow diagram of the study selection process.

The initial search identified 7054 articles excluding duplicates. After a total of 6894 papers were excluded during the title and abstract screening phases, the remaining 160 full texts were analysed. Out of these studies, 97 were excluded on the grounds of not meeting eligibility criteria, while 5 potentially eligible reports were not retrievable. Consequently, the remaining 58 articles from 57 studies were eligible for analysis, of which 54 studies could be included in the quantitative synthesis (Fig. 1). The list of studies that were not accessible and excluded at the full-text stage, with the primary reason for their exclusion, is available in Supplementary Data 4. The study characteristics of included studies are summarised in Supplementary Table S5. Kappa statistics indicated a good level of agreement during the abstract screening phase (kappa = 0.87) and the full-text analysis (kappa = 0.91).

**Fig. 2:**
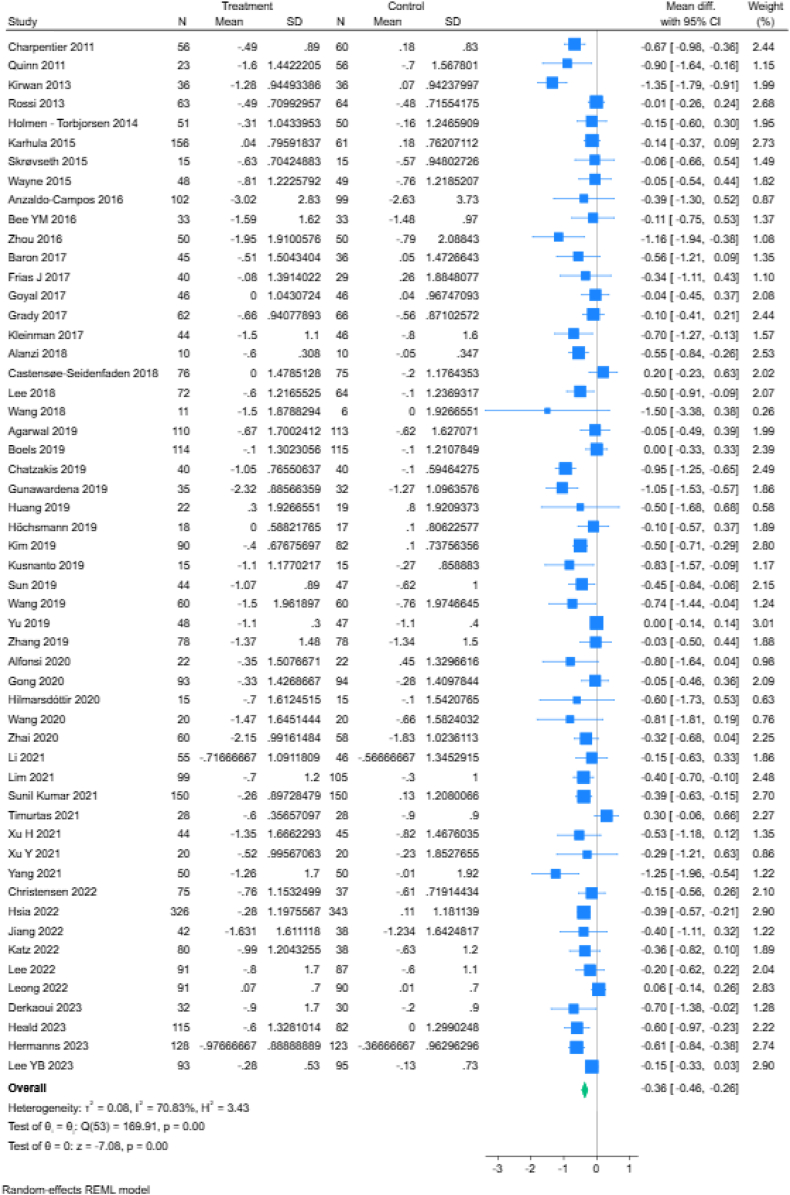
Meta-analysis of mobile app-based interventions versus control for improving glycaemic control. The size of each square indicates the weight of evidence from individual studies. Studies with confidence intervals (CIs) crossing zero (vertical line) are inconclusive; powerful studies (those with larger participant numbers and lower SDs) have narrower CIs; the diamond represents the summary effect size in the overall sample, with the width of the diamond indicating the 95% CI.

### Study and participant characteristics

The year of publication of trial reports ranged from 2011 to 2023, with a progressive increase in the number of studies per year and the apex reached in 2019, with 12 included publications. The mean (range) number of participants per trial was 144 (20–669). The length of the intervention period ranged from 12 weeks to 12 months, with most studies (29/57, 50.9%) testing the intervention over a 6-month period. Out of 57 studies, 43 (75.4%) were two-arm, while 10 (19.3%) were three-arm and 3 (5.3%) had four arms.^59^, ^60^, ^61^ Patients in the control group generally received usual care, while some studies introduced an active control, including a basic version of the app,^62^, ^63^, ^64^ additional advisory sessions at the study outset,^65^^,^^66^ or a dedicated exercise education program.^67^ In terms of target population, most studies addressed type 2 diabetes only (n = 40, 70.2%), whereas eleven studies exclusively focused on patients with type 1 diabetes (19.3%) and the remaining six enrolled both patients with type 1 and type 2 diabetes.^68^, ^69^, ^70^, ^71^, ^72^, ^73^ The majority of studies targeted adult populations, with wide age ranges covering the 16–80 years span. In contrast, five studies (8.8%) exclusively targeted children, adolescents and youth with diabetes,^74^, ^75^, ^76^, ^77^, ^78^ while a single study solely recruited older patients, setting a lower age boundary of 65 years for inclusion.^79^ In terms of baseline HbA1c required for participation, the majority of the included studies (n = 35, 61.4%) imposed a pre-specified HbA1c threshold as an eligibility criterion, typically targeting individuals with poorly controlled diabetes. As a result of these inclusion criteria, a total number of 7365 participants were included in the selected studies, with an average age of 50.5 and an average baseline HbA1c equal to 8.50% (range 6.54–11.05%). For type 1 diabetes, there were a total of 1412 participants, with a mean age of 30.6 years, an average baseline HbA1c of 8.81%, and 50.1% being male. On the contrary, participants with type 2 diabetes totalled 5953, exhibiting a significantly higher average age (55.5 years) with comparable baseline HbA1c values (8.39%) and a similar proportion of male participants (53.8%).

### Intervention characteristics

#### Grounding on behavioural theories and other features of app development

Eleven studies (19.3%) reported a theoretical basis and referenced behavioural theories that informed the design of the app. The most commonly adopted theory was the self-efficacy theory,^80^ according to which people's beliefs in their ability to perform specific behaviours influence their choices, efforts expended, task persistence and emotional reactions. Self-efficacy theory was specifically mentioned in the development of five apps.^68^^,^^81^, ^82^, ^83^, ^84^ The Transtheoretical Model (TTM) of Behaviour Change,^85^ which presents behaviour change through stages describing the individual's status along a continuum from pre-contemplation to action and maintenance, was instead applied by four studies.^86^, ^87^, ^88^, ^89^ As for other factors adopted during the development process, 21 studies (36.8%) incorporated the inputs of relevant stakeholders, primarily patients and healthcare professionals, into a user-centred design process based on iterative methods such as usability testing procedures, thereby leading to the final version of the app adopted during the trial.

#### Target behaviours and modes of intervention delivery

The app-based interventions targeted several of the self-care behaviours included in the ADCES7.^50^ “Monitoring” individual data was a targeted behaviour in all included studies, except for two.^75^^,^^90^ As for the other self-care behaviours, “Healthy Eating” with nutritional management was addressed in 42 studies (73.7% of included studies), “Being Active” was included in 40 interventions (70.2%) by fostering regular physical activity, and “Taking medication” was targeted by 23 studies (40.4%) that enabled recording or promoted medication taking. As for the method of intervention delivery, 42 studies (73.7%) requested participants to own a smartphone, thus adopting a BYOD approach. The remaining studies either provided participants with identical study devices (n = 13, 22.8%) or adopted a mixed strategy, supplying participants with a device when they did not own one (n = 2, 3.6%).^71^^,^^91^ The majority of interventions included some additional contribution by a healthcare professional compared to that provided to control arm participants (n = 35, 61.4%). HCP involvement typically occurred within the app itself or through an associated web portal where professionals could access registered data and initiate remote consultations with the patient (n = 26). Alternatively, the additional involvement could still be powered by app data but practically delivered through further technological services such as SMS,^92^ telephone calls and dedicated support channels.^81^^,^^88^^,^^93^, ^94^, ^95^ Finally, two studies provided participants in the intervention arm with additional HCP involvement unrelated to the digital technology.^83^^,^^96^ As for the level of technology automation, 21 studies (36.8%) adopted apps with some form of automatic support based on algorithms directed at adjusting the insulin dose,^69^^,^^76^^,^^93^^,^^95^^,^^97^, ^98^, ^99^, ^100^, ^101^ providing customized messages based on input data,^60^^,^^63^^,^^64^^,^^77^^,^^86^^,^^88^^,^^102^^,^^103^ modifying caloric intake and diet habits,^66^^,^^96^^,^^104^ or tailoring physical activity regimens.^65^

#### BCTs included

Across the 57 studies, the number of incremental BCTs included ranged from 3 to 16 (mean 9.07, SD 3.39). The most frequently coded BCTs were: “*feedback on outcomes of behaviour*” (*n* = 46, coded in 80.7% of the selected studies); “*instruction on how to perform the behaviour*” (*n* = 44, 77.2%); “*feedback on behaviour*” (*n* = 43, 75.4%); *“self-monitoring of behaviour”* (*n* = 40, 70.2%). Of the 93 BCTs included in the taxonomy, 60 were not tracked in any of the intervention descriptions examined, while 18 BCTs were coded in a number of studies between 10% and 90% of the total and were hence included in the quantitative analysis. Supplementary Figure S6 presents the prevalence of observed BCTs in the included studies. During the BCT identification process, agreement levels were moderate (kappa = 0.76). No correspondence was found between behavioural theories cited and BCTs identified.

### Risk of bias of included trials

A summary of the results of the quality assessment of individually-randomized studies is provided in Supplementary Figure S7. Concerns about the overall risk of bias were identified in 19 studies (34.5% of individually-randomized studies), while another 19 studies were judged to be at high risk of bias. Potential bias most frequently arose from the impossibility to blind participants and the consequent potential contamination effects, with 44 studies (80.0%) showing some concerns and 9 studies (16.4%) at high risk of bias due to deviations from the intended interventions. Additional concerns were related to missing outcome data coupled with inadequate statistical methods to eliminate potential bias (50.9% of studies were classified either at high risk or with some concerns) and unavailability or retrospective registration of pre-specified analysis plans (52.7% of studies were judged to have some concerns or be at high risk of bias in the selection of the reported results). As for the two included cluster randomized trials, there were some concerns about risk of bias for one^105^ and low risk for the other.^60^ Study-level details of the risk of bias assessment are included in Supplementary Figures S8 and S9.

### Effects of apps on glycaemic control

**Fig. 3:**
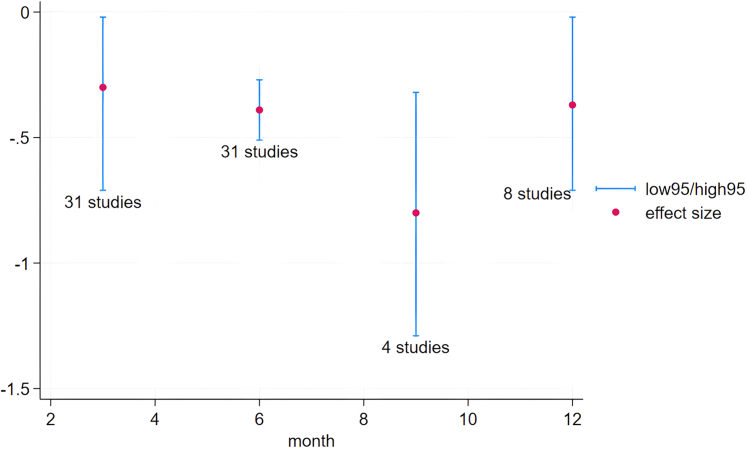
Temporal dynamics of the relationship between app interventions and glycaemic control.

Fifty-four studies had available data on effect sizes and were included in the quantitative synthesis. The meta-analysis main model presented in Fig. 2 identified a statistically significant reduction in HbA1c levels for intervention group participants compared to control arm individuals by −0.36 in mean difference (95% CI = −0.46 to −0.26, *p* < 0.001). There was substantial statistical heterogeneity between studies in effect size (*I*^*2*^ = 70.83%). After running the analysis at different time points including all studies with available effect size data (Fig. 3 and Supplementary Figures S10–S13), we observed that at 3 months, the decrease in HbA1c levels was equal to −0.31 (95% CI = −0.43 to −0.20, *p* < 0.001) with 34 studies reporting data at that time point, while at 6 months the overall effect size further improved to −0.38 (95% CI = −0.50 to −0.27, 32 studies, *p* < 0.001). Fewer studies reported outcome data at the 9-month (5 studies) and 12-month time points (9 studies), with an ameliorative impact of app usage on HbA1c levels respectively equal to −0.66 (95% CI = −1.09 to −0.24, *p* < 0.001) and −0.36 (95% CI = −0.65 to −0.06, *p* = 0.02) percentage points. Statistical heterogeneity was substantial at all considered time points.

Sixteen studies reported effect size data both 3 and 6 months after the beginning of the study. Pooling data across these studies confirmed the previous results, demonstrating a favourable improvement of app interventions between the 3-month (pooled mean difference = −0.34; 95% CI = −0.49 to −0.18, *p* < 0.001) and the 6-month time points (−0.46; 95% CI = −0.65 to −0.28, *p* < 0.001).

### Exploring heterogeneity of study results: meta-regression analyses

Subgroup analyses were initially conducted to explore potential sources of heterogeneity in the results. No between-group difference was observed between studies identifying glycaemic control as either a primary or secondary outcome. However, upon grounding interventions based on overall risk of bias, studies judged at high risk of bias showed a more favourable pooled effect (mean difference = −0.50, 95% CI = −0.69 to −0.31, *p* < 0.001) compared to the other two subgroups. Between-group differences were however not statistically significant. Instead, when interventions were grouped by study design, distinguishing between pilot and full-scale RCTs, a statistically significant group difference emerged (*p* = 0.04): the 46 full-scale RCTs showed a pooled effect equal to −0.33 (95% CI = −0.44 to −0.23, *p* < 0.001), while the 8 pilot RCTs had a more favourable effect of −0.63 (95% CI = −0.89 to −0.36, *p* < 0.001). To further explore the determinants of heterogeneity across studies and the moderating variables of effect size, we conducted univariate meta-regressions including the 18 BCTs that were coded in between 10% and 90% of the studies, along with four participant characteristics, two developmental features, three intervention specifications (the use of a study vis-à-vis a personal device, technology automation, and healthcare professional involvement), and the main behaviours targeted by the apps (healthy eating, being active, taking medication) as covariates (Table 1). Among the different BCTs examined, “*self-monitoring of behaviour*” explained the greatest amount of between-study variability (*R*^*2*^ = 20.1%) and was associated with a statistically significant beneficial change in glycaemic control (*β* = −0.30, *p* < 0.01). Additionally, “*self-monitoring outcome(s) of behaviour*” (*β* = −0.25, *p* = 0.02, *R*^*2*^ = 9.1%), “*user involvement*” as a developmental feature (*β* = 0.24, *p* = 0.02, *R*^*2*^ = 14.0%) and “*taking medication*” as a target behaviour (*β* = −0.25, *p* = 0.02, *R*^*2*^ = 5.2%) were identified as significant predictors of intervention effect sizes. Among the other variables, uncontrolled average HbA1c among study participants approached statistical significance (*β* = −0.19, *p* = 0.06, *R*^*2*^ = 8.4%), while the remaining ones had limited explanatory power.

**Table 1:** Univariate meta-regression analyses for selected study and intervention characteristics.

Model	Covariate	Type	β (95% CI)	*p*-value	R^2^ (%)
1	Male proportion	Participant characteristic	0.00 (0.00, 0.01)	0.261	0.67
2	Target population (type 1 diabetes)	Participant characteristic	−0.09 (−0.31, 0.14)	0.460	0
3	Target population (type 2 diabetes)	Participant characteristic	0.12 (−0.14, 0.38)	0.357	1.17
4	Uncontrolled HbA1c	Participant characteristic	−0.19 (−0.39, 0.00)	0.055	8.43
5	BC theories	Developmental feature	0.17 (−0.08, 0.43)	0.189	3.76
6	User involvement	Developmental feature	0.24 (0.04, 0.43)	0.020	14.00
7	Additional HCP involvement	Intervention characteristic	−0.01 (−0.22, 0.20)	0.949	0
8	Device type	Intervention characteristic	−0.06 (−0.31, 0.18)	0.617	0
9	Technology automation	Intervention characteristic	−0.13 (−0.33, 0.07)	0.199	5.20
10	Healthy eating	Target behaviour	−0.10 (−0.33, 0.12)	0.368	0
11	Being active	Target behaviour	0.08 (−0.14, 0.29)	0.488	0
12	Taking medication	Target behaviour	−0.25(−0.46, −0.05)	0.017	5.20
13	1.1 Goal setting (behavior)	BCTs	0.12 (−0.08, 0.33)	0.237	0
14	1.2 Problem solving	BCTs	0.08 (−0.24, 0.40)	0.605	0
15	1.3 Goal setting (outcome)	BCTs	0.00 (−0.20, 0.20)	0.995	0
16	1.4 Action planning	BCTs	−0.01 (−0.21, 0.20)	0.951	0
17	1.5 Review behavior goal(s)	BCTs	0.03 (−0.28, 0.35)	0.835	0
18	1.6 Discrepancy between current behavior and goal	BCTs	0.15 (−0.08, 0.38)	0.207	1.52
19	2.2 Feedback on behaviour	BCTs	−0.16 (−0.38, 0.07)	0.170	0.92
20	2.3 Self-monitoring of behaviour	BCTs	−0.30 (−0.50, −0.10)	0.003	20.08
21	2.4 Self-monitoring of outcome(s) of behaviour	BCTs	−0.25 (−0.48, −0.04)	0.016	9.11
22	2.6 Biofeedback	BCTs	0.14 (−0.07, 0.34)	0.185	0
23	2.7 Feedback on outcome(s) of behavior	BCTs	−0.18 (−0.43, 0.07)	0.154	4.50
24	3.1 Social support (unspecified)	BCTs	0.16 (−0.05, 0.37)	0.146	6.43
25	4.1 Instruction on how to perform the behavior	BCTs	0.00 (−0.24, 0.24)	0.978	0
26	5.1 Information about health consequences	BCTs	0.07 (−0.14, 0.27)	0.527	0
27	6.1 Demonstration of the behavior	BCTs	0.02 (−0.19, 0.24)	0.828	0
28	7.1 Prompts/cues	BCTs	−0.14 (−0.34, 0.06)	0.172	0.12
29	10.4 Social reward	BCTs	−0.08 (−0.34, 0.17)	0.527	0
30	12.5 Adding objects to the environment	BCTs	0.16 (−0.05, 0.36)	0.135	0.94
31	Total number of BCTs	BCTs	−0.00 (−0.03, 0.03)	0.998	0

Note: BC = behaviour change; BCT = behaviour change technique; HCP = healthcare professional.

In the subsequent multivariate model, which included only variables with a significant association with effect size in the univariate model, the *R*^*2*^ coefficient improved up to 25.9%. In this model, the BCT “*self-monitoring of behaviour*” (*β* = −0.22, *p* = 0.04) and “*taking medication*” (*β* = −0.20, *p* = 0.05) remained significantly associated with intervention effectiveness predicting more effective interventions (Table 2). The same meta-regression analyses were run after excluding the 8 pilot RCTs. The results of univariate models were strengthened in terms of heterogeneity explained by moderator variables, with the inclusion of BCT “*self-monitoring of behaviour*” exhibiting an *R*^*2*^ coefficient of 27.8% and a positive association with better performances of the intervention on glycaemic control (*β* = −0.34, *p* = 0.001). However, no variable was statistically significant in the multivariate meta-regression.

**Table 2:** Multivariate meta-regression analysis.

Covariate	Type	Classification	N	Effect size (95% CI)	*I*^*2*^	β (95% CI)	*p*-value
End user involvement	*Developmental feature*	Yes	21	−0.20 (−0.33, −0.06)	50%	0.14 (−0.07, 0.34)	0.184
		No	33	−0.45 (−0.57, −0.32)	73%		
2.3 Self-monitoring of behaviour	*BCT*	Yes	37	−0.46 (−0.57, −0.34)	68%	−0.22 (−0.44, −0.01)	0.044
		No	17	−0.15 (−0.30, 0.00)	59%		
2.4 Self-monitoring of outcome(s) of behaviour	*BCT*	Yes	35	−0.45 (−0.57, −0.33)	70%	−0.07 (−0.29, 0.15)	0.543
		No	19	−0.20 (−0.36, −0.05)	66%		
Taking medication	*Target behaviour*	Yes	22	−0.55 (−0.74, −0.35)	67%	−0.20 (−0.39, 0.00)	0.050
		No	32	−0.28 (−0.38, −0.17)	69%		

Note**:** N = number of studies.

### Sensitivity analyses and risk of reporting bias

The sensitivity analyses yielded consistent results with the primary meta-analysis, as the overall effect was not sensitive to the inclusion of individual studies, and the estimate of the reduction in HbA1c ranged from −0.37 to −0.33. The contour-enhanced funnel plot method was used to explore the presence of small-study effects (Supplementary Figure S14). The graph displayed some asymmetry, with less precise studies with higher standard errors reporting more favourable effect sizes towards app interventions either at the 1% or 5% level, compared to the more precise studies that more frequently reported nonsignificant results. The absence of small studies in the area of statistical nonsignificance might indicate potential publication bias, as confirmed by the Egger test (*p* < 0.01). Including the type of study design as a moderator increased the Egger test statistic but the regression remained statistically significant (*p* = 0.03), indicating that the presence of small-study effects may be partially attributable to heterogeneity induced by the inclusion of pilot RCTs but also dependent on publication bias. The trim-and-fill method identified six additional studies possibly missing; after imputing these studies, the updated estimate of the effect size would be −0.29 (95% CI = −0.40 to −0.18).

## Discussion

This systematic review of trials investigated the impact of behavioural interventions delivered via mobile apps on health outcomes of a highly prevalent NCD such as diabetes. Our search identified 57 randomized studies with diverse app-based interventions, ranging from virtual coaches and carbohydrate counting apps, to insulin dose calculators and solutions incorporating telehealth for data transmission.^106^ Overall, our findings confirmed that apps are effective in improving glycaemic control in patients with diabetes, with a pooled effect size of −0.36, a moderate result that is similar but slightly less favourable than estimates from previous meta-analyses covering both type 1 and type 2 diabetes.^32^^,^^39^ Our supplementary meta-analysis models make an additional contribution by offering novel estimates of the longitudinal effect of apps over time. Our data show a reversed bell-shaped curve, with a gradual improvement in app performance peaking around the nine-month time point, followed by a decrease in effectiveness thereafter. A similar trend was reported by Kebede et al. (2018), showing improved effect size estimates between 3 and 6 months, and decreasing at 9–12 months into the intervention.^46^ Other estimates have alternatively showed improved effectiveness with longer intervention periods,^102^ no significant differences between shorter (≤3 months) and longer (3–6 months) durations,^35^ or concluded that studies with a shorter follow-up duration (<6 months) displayed a larger (but nonsignificant) reduction compared to those with longer follow-ups.^36^^,^^38^ While previous estimates were primarily based on subgroup analyses, our approach considered all available data points at follow-up, allowing for multiple imputations for each study. Despite the variability in the number of observations at each time point, our models are based on a consistent number of studies and produced statistically significant pooled estimates at all time points. Furthermore, exclusively incorporating a homogeneous panel of 16 studies with available effect size data at both 3 and 6 months reaffirmed the pattern of improvement between these time periods, as the overall pooled effect increased from −0.31 to −0.38. This trend might signify a gradual learning effect or potentially indicate a temporal lag in the translation of the intervention impact on health outcomes, especially when moderated by prior effects on behavioural outcomes. However, it is essential to completement these analyses with data on app utilization, given the persisting challenge of sustained engagement. Empirical evidence shows a generic tendency towards discontinuation, with a systematic review reporting a pooled dropout rate of 43% in app-based interventions for chronic diseases.^107^ In a diabetes-focused observational study of a support app, the average 180-day user activity ratio, representing active days over potentially active ones, ranged from 0.05 to 0.55, with significant variability across app modules highlighting rapid discontinuation.^108^ Other studies consistently reported suboptimal compliance rates.^109^^,^^110^ Although our data hint at a potential temporal trend in app effectiveness for this specific population, the absence of information on app usage limits our ability to contribute to understanding the nuanced, potentially non-linear relationship between quantitative engagement and app effectiveness, as effective engagement is necessarily subjective and varies individually. We also analysed the intervention content to identify characteristics associated with mobile app effectiveness and to explain the substantial heterogeneity between studies observed through meta-analyses. We used a well-established taxonomy to classify intervention content and supplemented it with considerations related to features of the development processes and other intervention design characteristics that we had already outlined in a previous work, which focused on app interventions across various NCDs.^111^ The univariate meta-regression models indicated that the number of BCTs was not associated with improved effectiveness, emphasizing the importance of quality and combination of the right techniques. Despite previous meta-regressions in different domains alternatively demonstrated a lack^45^^,^^46^^,^^57^ or presence^112^^,^^113^ of a positive association between the number of BCTs and study effectiveness, all studies have underscored the necessity for further investigation on this matter. According to our analyses, improvements in HbA1c were instead associated with individual BCTs, with “*self-monitoring of behaviour*” explaining the greatest amount of heterogeneity (R^2^ = 20.08%). The related subgroup analysis showed that the 17 studies not adopting this technique produced a pooled effect size of −0.15 (95% CI = −0.30 to 0.00), while the 37 employing it generated a more significant reduction (−0.46, 95% CI = −0.57 to −0.34) that is also clinically significant, as a decrease in HbA1c by 0.3 percentage points or more is identified as the threshold to reduce diabetic complications.^112^, ^113^, ^114^ Other moderating variables associated with a statistically significant impact on effect size were the BCT “*self-monitoring of outcome(s) of behaviour*”, “*taking medication*” as a target behaviour, and “*user involvement*” among development features. Incorporating all statistically significant variables into a multivariate model further enhanced the proportion of accounted variance attributed to observed differences in study intervention design, reaching up to 25.88%. In this model, the inclusion of “*self-monitoring of behaviour*” as BCT and the focus on taking medication as a target behaviour were significantly associated with intervention effectiveness (*p* ≤ 0.05), while user involvement and “*self-monitoring of outcomes of behaviour*” were no longer statistically significant.

Self-monitoring has been recognized as an effective component for tracking behaviour change and enabling self-regulation across various meta-regressions targeting different behaviours.^57^^,^^115^, ^116^, ^117^ Similar results emerged from meta-regressions analysing the impact of DBCIs in diabetes, which identified an association between the inclusion of self-monitoring of outcomes and statistically significant reductions in HbA1c.^45^^,^^46^ Our results confirm that actively tracking individual parameters and data using DBCIs can enable patients to take control of their condition and enhance emotional investment, a crucial component of self-management.^118^ Based on our analyses, self-monitoring emerges as a more effective strategy compared to passive sensing, which captures data about a person without requiring any extra effort on their part, confirming the importance of self-reporting when DBCIs are intended to support behaviour change.^119^

However, a different meta-analysis focusing solely on interventions targeting diet and physical activity in type 2 diabetes through in-person individual or group sessions reached quite diverse conclusions, showing that BCTs linked with clinically meaningful improvements in glycaemic control were “*action planning*”, “*instruction on how to perform a behaviour*”, “*behavioural practice/rehearsal*”, and “*demonstration of the behaviour*”.^113^ These findings reinforce the notion that individual BCTs and their groupings may have varying effectiveness depending on the mode of delivery: while training and demonstration might be effective in traditional face-to-face interventions, other techniques such as self-monitoring may be required for DBCIs targeting self-management.

Adherence to medication remains a critical problem for NCDs, particularly for diabetes. A systematic review of 27 studies reported medication adherence rates ranging from 38.5% to 93.1% in type 2 diabetes,^120^ identifying several modifiable factors such as health beliefs, health literacy, and early nonpersistence as key targets to optimize diabetes control and slow its progression.^121^ Mobile apps can contribute to improved medication adherence through multiple behavioural channels, including the provision of accurate medication lists, reminder prompts, and integration of medication regimens with food and blood glucose levels.^50^ Coherently, a large proportion of diabetes self-management apps available in public stores featured elements to enhance medication adherence, employing a mix of educational, behavioural and affective strategies.^122^ While a prior study revealed a moderate and significant effect of mobile apps on medication adherence in type 2 diabetes,^123^ it was beyond our scope to provide a quantitative estimate of this relationship. Nonetheless, our study confirmed that apps for diabetes self-management are particularly effective in influencing clinical outcomes when they target medication adherence: the 22 app-based interventions focusing on medication adherence as a target behaviour showed a pooled effect of −0.55 (95% CI = −0.74 to −0.35), in contrast to the remaining 32 interventions which produced a cumulative beneficial effect on glycaemic control of −0.28 (95% CI = −0.38 to −0.17).

User involvement in the design of DBCIs was statistically associated with effect size with a positive *β* coefficient, indicating that interventions incorporating this development feature were predictive of improved glycaemic control (with a pooled effect size of −0.20), but less effective compared to studies without it. While no longer significant in the multivariate model when accounting for the other main characteristics associated with effect size, this finding warrants careful consideration, highlighting that the inability to navigate the practical challenges in implementing user-centred design (UCD) may limit its beneficial impact.^124^

No other variable demonstrated a differential impact on app effectiveness, including diabetes type, except for study design: pilot and feasibility RCTs exhibited a more favourable pooled effect than full-scale studies, confirming that larger trials are often associated with smaller, yet closer to the to-be-expected true effects.^125^

### Outstanding questions

Our extensive quantitative analysis warrants additional investigation into the mechanisms underpinning the observed associations. In particular, a more significant contribution is anticipated from behavioural theories, which are essential for effective development of DBCIs.^41^^,^^126^ Only a restricted subset of trials integrated behaviour change theories, with even fewer offering a comprehensive account of the role of theories in the actual development of apps. In coherence, a majority of the BCTs outlined in the taxonomy were notably absent from the intervention descriptions in the selected studies, highlighting the current challenge in harnessing specific channels to activate effective self-management. These findings align with previous systematic reviews on mobile apps for NCDs,^45^^,^^111^^,^^113^ revealing unexplored areas that warrant investigation for better design and testing of app-based DBCIs.

In terms of their design, DBCIs not only exhibit a lack of theory integration but, even when theory-embedded, often rely on a limited number of classical theories exclusively.^127^ Traditional theories may prove inadequate in the digital age because of their inability to consider the temporal aspect of behaviour change and the possibility to adapt decision-making.^128^ Most of the current behaviour change theories are static and have been conceptualized based on group-level differences rather than change within single individuals.^40^ On the other hand, DBCIs possess inherent characteristics that render them unique^129^ and allow them to increasingly utilize personal information to continuously adapt provision of support to ever-changing individual needs.^130^ Moreover, compared to traditional interventions, DBCIs result in significantly more variation in individuals’ exposure to their various components and the different BCTs they include.^17^ This is due to users often having the flexibility to choose their preferred engagement modalities. The proliferation of DBCIs targeting self-management of NCDs, particularly diabetes, along with the advancements in recording and tracking “digital traces”, remarkably expands the opportunities to empirically test and advance the understanding of human behaviour using new theoretical frameworks in real-world setting.^40^ This not only holds the potential for a positive impact on public health,^131^ but also underscores the necessity to link individual BCTs with the mechanisms of action through which they generate their effects.^132^ A multidisciplinary effort that cross-fertilizes psychology and behaviour change theoretical paradigms with medicine and social science is hence imperative for the effective advancement of digital solutions based on behaviour change theories. Contextually, advancement is necessary in the evidence generation process. Current studies frequently face methodological quality issues, as highlighted by our risk of bias assessment. Additionally, they rarely include personalization features, as they adhere to traditional RCT designs that offer standardized interventions to all participants, irrespective of individual characteristics, preferences and life context. Although our inclusion criteria allowed for the consideration of more flexible and agile methodologies,^133^^,^^134^ adaptive designs remain underutilized in the development and testing of digital interventions, and no study with an adaptive design met all our inclusion criteria.

### Strengths and limitations

This study, drawing from over 50 trials, employed rigorous methodologies adhering to the PRISMA and Cochrane guidelines for conducting meta-analyses. Both univariate and multivariate meta-regressions were used to scrutinize evidence, aiming to identify BCTs and other intervention characteristics that can help explain the large heterogeneity observed in the review. However, there are equally important considerations that our study could not address and a number of limitations must be acknowledged. First, this review lacked adequate power to concomitantly test numerous variables and could not factor in a relevant number of undocumented variables. Second, we did not specifically search databases like PsycInfo, although most of its indexed journals are included in other databases we searched. Third, the classification of intervention content was necessarily arbitrary, due to the lack of consistent definitions and standardized assessments for the optimal categorization of digital interventions. Additionally, the taxonomy used for the BCT analysis was not specifically developed for mobile apps, inevitably entailing authors’ judgement. The coding of BCT and behaviour change theories was solely reliant on information provided in the study report and related sources. Some of the features may have gone untracked, possibly resulting in an underestimation of the number of BCTs tapped and theories employed. Regarding the theoretical basis, ascertaining actual fidelity to the cited theory was challenging, as the necessary information for such assessment was consistently unreported.

### Conclusions

Our systematic review and meta-analysis provide compelling evidence supporting the efficacy of apps in diabetes self-management for both type 1 and type 2 diabetes, identifying characteristics of these interventions that statistically correlate with effect size. These results offer valuable recommendations for future research and practice, guiding the development of more effective interventions. As the prevalence of NCDs like diabetes is expected to alarmingly increase in the coming years,^4^ the future sustainability of healthcare systems will heavily rely on their capacity to keep people healthy as long as possible and cost-effectively manage chronic conditions.^135^ Until now, DBCIs for diabetes self-management have inadequately incorporated essential inputs, behavioural theories, and BCTs for their effective design. However, in the current data-rich science environment, there are several opportunities to improve self-management by combining DBCIs with necessary progress in behavioural theories and advancements in study designs used to generate evidence.

## Contributors

RT primarily conceptualized and designed the study, with support from FP, MC, and OC. FP and LS were responsible for database search and data extraction. Risk of bias assessment for the included studies was performed by FP, OC and LS, who also accessed and analysed the underlying data, taking responsibility for the findings presented in the manuscript. All authors collectively interpreted the results, drafted the manuscript, and have reviewed and approved its final version.

## Data sharing statement

The data collected for this study will be provided upon request to the corresponding author.

## Declaration of interests

The authors declare no competing interests.
