# Supplementary material for: Which behaviour change techniques work best for diabetes self-management mobile apps? Results from a systematic review and meta-analysis of randomised controlled trials
Source: eBioMedicine. 2024 Apr 5;103:105091. doi: 10.1016/j.ebiom.2024.105091 (PMC11002812; doi:10.1016/j.ebiom.2024.105091)

**Which behaviour change techniques work best for diabetes self-management mobile apps? Results from a systematic-review and meta-analysis of randomised controlled trials**

Rosanna Tarricone^1,2^, Francesco Petracca^2^, Liv Svae^2^, Maria Cucciniello^1,2^, and Oriana Ciani^2^

1. Department of Social and Political Sciences, Bocconi University, Milan, Italy
2. Centre for Research on Health and Social Care Management (CERGAS), Government, Health and Not for Profit Division, SDA Bocconi School of Management, Milan, Italy

**Supplementary material**

[**Supplementary Data 1 – PRISMA checklist** 2](#_Toc161330814)

[**Supplementary Data 2 – Search strategy** 5](#_Toc161330815)

[**Supplementary Data 3 – Methodological details for the estimation of effect size when median and interquartile ranges are reported** 6](#_Toc161330816)

[**Supplementary Data 4 – List of studies excluded at full-text stage with primary reason for exclusion and inaccessible studies** 7](#_Toc161330817)

[**Supplementary Table 5 – Study and sample characteristics of included studies** 14](#_Toc161330818)

[**Supplementary Figure 6 – Prevalence of Behaviour Change Techniques in selected app-based DBCIs** 21](#_Toc161330819)

[**Supplementary Figure 7 – Risk of Bias for HbA1c changes: summary plot for individually-randomized studies (N=55)** 22](#_Toc161330820)

[**Supplementary Figure 8 – Study-level details of the risk of bias assessment for individually-randomized studies** 23](#_Toc161330821)

[**Supplementary Figure 9 – Study-level details of the risk of bias assessment for cluster-randomized studies** 24](#_Toc161330822)

[**Supplementary Figure 10 – Meta-analysis of effect size at the 3-month time point (n=34 studies)** 25](#_Toc161330823)

[**Supplementary Figure 11 – Meta-analysis of effect size at the 6-month time point (n=32 studies)** 26](#_Toc161330824)

[**Supplementary Figure 12 – Meta-analysis of effect size at the 9-month time point (n=5 studies)** 27](#_Toc161330825)

[**Supplementary Figure 13 – Meta-analysis of effect size at the 12-month time point (n=9 studies)** 28](#_Toc161330826)

[**Supplementary Figure 14 – Contour-enhanced funnel plot to detect small-study effects** 29](#_Toc161330827)

# **Supplementary Data 1 – PRISMA checklist**

| **Section and Topic** | **Item #** | **Checklist item** | **Location where item is reported** |
| --- | --- | --- | --- |
| **TITLE** | | |  |
| Title | 1 | Identify the report as a systematic review. | Title Page – Page 1 |
| **ABSTRACT** | | |  |
| Abstract | 2 | See the PRISMA 2020 for Abstracts checklist. | Page 2 |
| **INTRODUCTION** | | |  |
| Rationale | 3 | Describe the rationale for the review in the context of existing knowledge. | Introduction – Page 4-5 |
| Objectives | 4 | Provide an explicit statement of the objective(s) or question(s) the review addresses. | Introduction – Page 5 |
| **METHODS** | | |  |
| Eligibility criteria | 5 | Specify the inclusion and exclusion criteria for the review and how studies were grouped for the syntheses. | Methods – Page 6 |
| Information sources | 6 | Specify all databases, registers, websites, organisations, reference lists and other sources searched or consulted to identify studies. Specify the date when each source was last searched or consulted. | Methods – Page 6 |
| Search strategy | 7 | Present the full search strategies for all databases, registers and websites, including any filters and limits used. | Supplementary Data 2 |
| Selection process | 8 | Specify the methods used to decide whether a study met the inclusion criteria of the review, including how many reviewers screened each record and each report retrieved, whether they worked independently, and if applicable, details of automation tools used in the process. | Methods – Page 6-7 |
| Data collection process | 9 | Specify the methods used to collect data from reports, including how many reviewers collected data from each report, whether they worked independently, any processes for obtaining or confirming data from study investigators, and if applicable, details of automation tools used in the process. | Methods – Page 6-7 |
| Data items | 10a | List and define all outcomes for which data were sought. Specify whether all results that were compatible with each outcome domain in each study were sought (e.g. for all measures, time points, analyses), and if not, the methods used to decide which results to collect. | Methods – Page 7-8 |
|  | 10b | List and define all other variables for which data were sought (e.g. participant and intervention characteristics, funding sources). Describe any assumptions made about any missing or unclear information. | Methods – Page 7-8 |
| Study risk of bias assessment | 11 | Specify the methods used to assess risk of bias in the included studies, including details of the tool(s) used, how many reviewers assessed each study and whether they worked independently, and if applicable, details of automation tools used in the process. | Methods – Page 8 |
| Effect measures | 12 | Specify for each outcome the effect measure(s) (e.g. risk ratio, mean difference) used in the synthesis or presentation of results. | Methods – Page 7 |
| Synthesis methods | 13a | Describe the processes used to decide which studies were eligible for each synthesis (e.g. tabulating the study intervention characteristics and comparing against the planned groups for each synthesis (item #5)). | Methods – Page 9-10 |
|  | 13b | Describe any methods required to prepare the data for presentation or synthesis, such as handling of missing summary statistics, or data conversions. | Methods – Page 9-10 |
|  | 13c | Describe any methods used to tabulate or visually display results of individual studies and syntheses. | Methods – Page 9-10 |
|  | 13d | Describe any methods used to synthesize results and provide a rationale for the choice(s). If meta-analysis was performed, describe the model(s), method(s) to identify the presence and extent of statistical heterogeneity, and software package(s) used. | Methods – Page 9-10 |
|  | 13e | Describe any methods used to explore possible causes of heterogeneity among study results (e.g. subgroup analysis, meta-regression). | Methods – Page 9-10 |
|  | 13f | Describe any sensitivity analyses conducted to assess robustness of the synthesized results. | Methods – Page 9-10 |
| Reporting bias assessment | 14 | Describe any methods used to assess risk of bias due to missing results in a synthesis (arising from reporting biases). | Methods – Page 9-10 |
| Certainty assessment | 15 | Describe any methods used to assess certainty (or confidence) in the body of evidence for an outcome. | N/A |
| **RESULTS** | | |  |
| Study selection | 16a | Describe the results of the search and selection process, from the number of records identified in the search to the number of studies included in the review, ideally using a flow diagram. | Results – Page 10 |
|  | 16b | Cite studies that might appear to meet the inclusion criteria, but which were excluded, and explain why they were excluded. | Supplementary Data 3 |
| Study characteristics | 17 | Cite each included study and present its characteristics. | Supplementary Table 4 |
| Risk of bias in studies | 18 | Present assessments of risk of bias for each included study. | Results – Page 12  Supplementary Figure 6-8 |
| Results of individual studies | 19 | For all outcomes, present, for each study: (a) summary statistics for each group (where appropriate) and (b) an effect estimate and its precision (e.g. confidence/credible interval), ideally using structured tables or plots. | Supplementary Table 4 |
| Results of syntheses | 20a | For each synthesis, briefly summarise the characteristics and risk of bias among contributing studies. | Results – Page 13 |
|  | 20b | Present results of all statistical syntheses conducted. If meta-analysis was done, present for each the summary estimate and its precision (e.g. confidence/credible interval) and measures of statistical heterogeneity. If comparing groups, describe the direction of the effect. | Figure 3-4  Supplementary Figure 9-12 |
|  | 20c | Present results of all investigations of possible causes of heterogeneity among study results. | Results – Page 13-14  Table 2 (?) |
|  | 20d | Present results of all sensitivity analyses conducted to assess the robustness of the synthesized results. | Results – Page 14 |
| Reporting biases | 21 | Present assessments of risk of bias due to missing results (arising from reporting biases) for each synthesis assessed. | Results – Page 14  Supplementary Figure 13 |
| Certainty of evidence | 22 | Present assessments of certainty (or confidence) in the body of evidence for each outcome assessed. | N/A |
| **DISCUSSION** | | |  |
| Discussion | 23a | Provide a general interpretation of the results in the context of other evidence. | Discussion – Page 15-18 |
|  | 23b | Discuss any limitations of the evidence included in the review. | Discussion – Page 18-19 |
|  | 23c | Discuss any limitations of the review processes used. | Discussion – Page 18-19 |
|  | 23d | Discuss implications of the results for practice, policy, and future research. | Discussion – Page 17-19 |
| **OTHER INFORMATION** | | |  |
| Registration and protocol | 24a | Provide registration information for the review, including register name and registration number, or state that the review was not registered. | Methods – Page 6 |
|  | 24b | Indicate where the review protocol can be accessed, or state that a protocol was not prepared. | Methods – Page 6 |
|  | 24c | Describe and explain any amendments to information provided at registration or in the protocol. | N/A |
| Support | 25 | Describe sources of financial or non-financial support for the review, and the role of the funders or sponsors in the review. | Acknowledgments – Page 20 |
| Competing interests | 26 | Declare any competing interests of review authors. | Declaration of interests – Page 20 |
| Availability of data, code and other materials | 27 | Report which of the following are publicly available and where they can be found: template data collection forms; data extracted from included studies; data used for all analyses; analytic code; any other materials used in the review. | Data sharing statement – Page 20 |

# **Supplementary Data 2 – Search strategy**

The detailed search strategies employed for combining the concepts of mobile app and diabetes are presented below.

**PubMed search**

| **Mobile apps** [mobile health [Title/Abstract] OR mhealth [Title/Abstract] OR m-health [Title/Abstract] OR mobile phone* [Title/Abstract] OR smartphone* [Title/Abstract] OR health app* [Title/Abstract] OR mobile app* [Title/Abstract] OR mobile applications [MeSH Terms] OR telemedicine [MeSH Terms]] |
| --- |
| **Diabetes** [diabetes* [Title/Abstract] OR t2dm [Title/Abstract] OR t1dm [Title/Abstract] OR iddm [Title/Abstract] OR niddm [Title/Abstract] OR dm [Title/Abstract] OR t1d [Title/Abstract] OR t2d [Title/Abstract] OR mody [Title/Abstract]] |

**Embase and Cochrane Central Register of Controlled Trials search**

| **#** | **Search** |
| --- | --- |
| **1** | mobile health OR mhealth OR m-health OR mobile phone* OR smartphone* OR mobile app* OR health app* |
| **2** | telemedicine OR telehealth |
| **3** | 1 OR 2 |
| **4** | diabetes OR diabetes mellitus OR DM OR type 1 diabetes OR type 1 diabetes mellitus OR t1dm OR T1D OR IDDM OR type 2 diabetes OR type 2 diabetes mellitus OR t2dm OR T2D OR NIIDM OR MODY |
| **5** | 3 AND 4 |
| **6** | Limit 5 to English language |
| **7** | Limit 6 to yr=“2008-Current” |

**Scopus search**

(TITLE-ABS-KEY (mobile health OR mhealth OR m-health OR mobile phone* OR smartphone* OR mobile app* OR health app*)) AND (TITLE-ABS-KEY (diabetes OR diabetes mellitus OR DM OR type 1 diabetes OR type 1 diabetes mellitus OR t1dm OR T1D OR IDDM OR type 2 diabetes OR type 2 diabetes mellitus OR t2dm OR T2D OR NIIDM OR MODY)) AND (LIMIT-TO ( PUBYEAR , 2023 ) OR LIMIT-TO ( PUBYEAR , 2022 ) OR LIMIT-TO ( PUBYEAR , 2021 ) OR LIMIT-TO ( PUBYEAR , 2020 ) OR LIMIT-TO ( PUBYEAR , 2019 ) OR LIMIT-TO ( PUBYEAR , 2018 ) OR LIMIT-TO ( PUBYEAR , 2017 ) OR LIMIT-TO ( PUBYEAR , 2016 ) OR LIMIT-TO ( PUBYEAR , 2015 ) OR LIMIT-TO ( PUBYEAR , 2014) OR LIMIT-TO ( PUBYEAR , 2013) OR LIMIT-TO ( PUBYEAR , 2012 ) OR LIMIT-TO ( PUBYEAR , 2011 ) OR LIMIT-TO ( PUBYEAR , 2010 ) OR LIMIT-TO ( PUBYEAR , 2009) OR LIMIT-TO ( PUBYEAR , 2008 ))

# **Supplementary Data 3 – Methodological details for the estimation of effect size when median and interquartile ranges are reported**

For studies providing medians and inter-quartile ranges only, Wan et al. (2014) propose the following estimation of sample mean and standard deviation.

$\bar{x}$ ≈ $\frac{q1+m+q3}{3}$

*S* ≈ $\frac{q3-q1}{\eta(n)}$

where:

*q*1 = the first quartile

*m* = the median

*q*3 = the third quartile

*n* = the sample size

*η(n)* = a function of *n,* whose value converges to 1.35 with sufficiently large *n*

# **Supplementary Data 4 – List of studies excluded at full-text stage with primary reason for exclusion and inaccessible studies**

***Ineligible intervention***

- *Not a mobile app or an intervention which can be alternatively delivered via app or other means*

Amante DJ, Harlan DM, Lemon SC, et al. Evaluation of a Diabetes Remote Monitoring Program Facilitated by Connected Glucose Meters for Patients With Poorly Controlled Type 2 Diabetes: Randomized Crossover Trial. JMIR Diabetes. 2021;6(1):e25574.

Anthony CA, Femino JE, Miller AC, et al. Diabetic Foot Surveillance Using Mobile Phones and Automated Software Messaging, a Randomized Observational Trial. Iowa Orthop J. 2020;40(1):35-42.

Azelton KR, Crowley AP, Vence N, et al. Digital Health Coaching for Type 2 Diabetes: Randomized Controlled Trial of Healthy at Home. Front Digit Health. 2021;3:764735.

Banu B, Ko KC, Khan MMH, Ali L, Barnighausen T, Sauerborn R, Souares A. Effects of traditional versus m-Health educational interventions for diabetic patients: a randomised controlled trial in peripheral district of Bangladesh. Diabetes Epidemiology and Management. 2023;9, 100106.

Capozza K, Woolsey S, Georgsson M, et al. Going mobile with diabetes support: a randomized study of a text message-based personalized behavioral intervention for type 2 diabetes self-care. Diabetes Spectr. 2015;28(2):83-91.

Crowley MJ, Tarkington PE, Bosworth HB, et al. Effect of a Comprehensive Telehealth Intervention vs Telemonitoring and Care Coordination in Patients With Persistently Poor Type 2 Diabetes Control: A Randomized Clinical Trial. JAMA Intern Med. 2022;182(9):943-952.

Egede LE, Dawson AZ, Walker RJ, Garraci E, Knapp RG. Randomized controlled trial of technology-assisted case management in low-income adults with type 2 diabetes: Effect on quality of life and blood pressure. J Telemed Telecare. 2021;1357633X211028491.

Esferani SV, Naghizadeh E, Albokordi M, Zakerkish M, Araban M. Effectiveness of a mobile-based educational intervention on self-care activities and glycemic control among the elderly with type 2 diabetes in southwest of Iran in 2020. Arch Public Health. 2022;80(1):201.

Fountoulakis S, Papanastasiou L, Gryparis A, Markou A, Piaditis G. Impact and duration effect of telemonitoring on ΗbA1c, BMI and cost in insulin-treated Diabetes Mellitus patients with inadequate glycemic control: A randomized controlled study. Hormones (Athens). 2015;14(4):632-643.

Franco DW, Alessi J, de Carvalho TR, et al. The impact of a telehealth intervention on the metabolic profile of diabetes mellitus patients during the COVID-19 pandemic - A randomized clinical trial. Prim Care Diabetes. 2022;16(6):745-752.

Gerber BS, Biggers A, Tilton JJ, et al. Mobile Health Intervention in Patients With Type 2 Diabetes: A Randomized Clinical Trial. JAMA Netw Open. 2023;6(9):e2333629.

Istepanian RS, Zitouni K, Harry D, et al. Evaluation of a mobile phone telemonitoring system for glycaemic control in patients with diabetes. J Telemed Telecare. 2009;15(3):125-128.

Jia W, Zhang P, Zhu D, et al. Evaluation of an mHealth-enabled hierarchical diabetes management intervention in primary care in China (ROADMAP): A cluster randomized trial. PLoS Med. 2021;18(9):e1003754.

Kang J, Chen Y, Zhao Y, Zhang C. Effect of remote management on comprehensive management of diabetes mellitus during the COVID-19 epidemic. Prim Care Diabetes. 2021;15(3):417-423.

Kardas P, Lewandowski K, Bromuri S. Type 2 Diabetes Patients Benefit from the COMODITY12 mHealth System: Results of a Randomised Trial. J Med Syst. 2016;40(12):259.

Kerfoot BP, Gagnon DR, McMahon GT, Orlander JD, Kurgansky KE, Conlin PR. A Team-Based Online Game Improves Blood Glucose Control in Veterans With Type 2 Diabetes: A Randomized Controlled Trial. Diabetes Care. 2017;40(9):1218-1225.

Kim HS, Sun C, Yang SJ, et al. Randomized, Open-Label, Parallel Group Study to Evaluate the Effect of Internet-Based Glucose Management System on Subjects with Diabetes in China. Telemed J E Health. 2016;22(8):666-674.

Kleinman NJ, Shah A, Shah S, Phatak S, Viswanathan V. Improved Medication Adherence and Frequency of Blood Glucose Self-Testing Using an m-Health Platform Versus Usual Care in a Multisite Randomized Clinical Trial Among People with Type 2 Diabetes in India. Telemed J E Health. 2017;23(9):733-740.

María Gómez A, Cristina Henao D, León Vargas F, et al. Efficacy of the mHealth application in patients with type 2 diabetes transitioning from inpatient to outpatient care: A randomized controlled clinical trial. Diabetes Res Clin Pract. 2022;189:109948.

McLeod M, Stanley J, Signal V, et al. Impact of a comprehensive digital health programme on HbA1c and weight after 12 months for people with diabetes and prediabetes: a randomised controlled trial. Diabetologia. 2020;63(12):2559-2570.

Myers A, Presswala L, Bissoonauth A, et al. Telemedicine for Disparity Patients With Diabetes: The Feasibility of Utilizing Telehealth in the Management of Uncontrolled Type 2 Diabetes in Black and Hispanic Disparity Patients; A Pilot Study. J Diabetes Sci Technol. 2021;15(5):1034-1041.

Nayak A, Vakili S, Nayak K, et al. Use of Voice-Based Conversational Artificial Intelligence for Basal Insulin Prescription Management Among Patients With Type 2 Diabetes: A Randomized Clinical Trial. JAMA Netw Open. 2023;6(12):e2340232.

Patnaik L, Panigrahi SK, Sahoo AK, Mishra D, Beura S, Muduli AK. Mobile health application based intervention for improvement of quality of life among newly diagnosed type 2 diabetes patients. Clinical Diabetology; 2021;10(3), 276-283.

Poppe L, De Bourdeaudhuij I, Verloigne M, et al. Efficacy of a Self-Regulation-Based Electronic and Mobile Health Intervention Targeting an Active Lifestyle in Adults Having Type 2 Diabetes and in Adults Aged 50 Years or Older: Two Randomized Controlled Trials. J Med Internet Res. 2019;21(8):e13363.

Prabhakaran D, Jha D, Prieto-Merino D, et al. Effectiveness of an mHealth-Based Electronic Decision Support System for Integrated Management of Chronic Conditions in Primary Care: The mWellcare Cluster-Randomized Controlled Trial. Circulation. 2019;139(3):380-391.

Pyatak EA, Ali A, Khurana AR, et al. Research design and baseline participant characteristics of the Resilient, Empowered, Active Living with Diabetes - Telehealth (REAL-T) Study: A randomized controlled trial for young adults with type 1 diabetes. Contemp Clin Trials. 2023;135:107386.

Quinn CC, Clough SS, Minor JM, Lender D, Okafor MC, Gruber-Baldini A. WellDoc mobile diabetes management randomized controlled trial: change in clinical and behavioral outcomes and patient and physician satisfaction. Diabetes Technol Ther. 2008;10(3):160-168.

Ravari A, Sheikhoshaqi A, Mirzaei T, Raeisi M, Hassanshahi E, Kamiab Z. Effect of Tele-nursing on Blood Glucose Control among the Elderly with Diabetes: A Randomized Controlled Trial. Evidence Based Care. 2021;11(2), 54-63.

Signal V, McLeod M, Stanley J, et al. A Mobile- and Web-Based Health Intervention Program for Diabetes and Prediabetes Self-Management (BetaMe/Melon): Process Evaluation Following a Randomized Controlled Trial. J Med Internet Res. 2020;22(12):e19150.

Vaughan EM, Hyman DJ, Naik AD, Samson SL, Razjouyan J, Foreyt JP. A Telehealth-supported, Integrated care with CHWs, and MEdication-access (TIME) Program for Diabetes Improves HbA1c: a Randomized Clinical Trial. J Gen Intern Med. 2021;36(2):455-463.

Waki K, Fujita H, Uchimura Y, et al. DialBetics: A Novel Smartphone-based Self-management Support System for Type 2 Diabetes Patients. J Diabetes Sci Technol. 2014;8(2):209-215.

Yasmin F, Nahar N, Banu B, Ali L, Sauerborn R, Souares A. The influence of mobile phone-based health reminders on patient adherence to medications and healthy lifestyle recommendations for effective management of diabetes type 2: a randomized control trial in Dhaka, Bangladesh. BMC Health Serv Res. 2020;20(1):520.

Yin W, Liu Y, Hu H, Sun J, Liu Y, Wang Z. Telemedicine management of type 2 diabetes mellitus in obese and overweight young and middle-aged patients during COVID-19 outbreak: A single-center, prospective, randomized control study. PLoS One. 2022;17(9):e0275251.

- *App only a subsidiary element of a complex multifactorial intervention*

Alonso-Domínguez R, García-Ortiz L, Patino-Alonso MC, Sánchez-Aguadero N, Gómez-Marcos MA, Recio-Rodríguez JI. Effectiveness of A Multifactorial Intervention in Increasing Adherence to the Mediterranean Diet among Patients with Diabetes Mellitus Type 2: A Controlled and Randomized Study (EMID Study). Nutrients. 2019;11(1):162.

Alonso-Domínguez R, Patino-Alonso MC, Sánchez-Aguadero N, García-Ortiz L, Recio-Rodríguez JI, Gómez-Marcos MA. Effect of a multifactorial intervention on the increase in physical activity in subjects with type 2 diabetes mellitus: a randomized clinical trial (EMID Study). Eur J Cardiovasc Nurs. 2019;18(5):399-409.

Bender MS, Cooper BA, Park LG, Padash S, Arai S. A Feasible and Efficacious Mobile-Phone Based Lifestyle Intervention for Filipino Americans with Type 2 Diabetes: Randomized Controlled Trial [published correction appears in JMIR Diabetes. 2018 Dec 21;3(4):e12784]. JMIR Diabetes. 2017;2(2):e30.

Callan JA, Sereika SM, Cui R, et al. Cognitive Behavioral Therapy (CBT) Telehealth Augmented With a CBT Smartphone Application to Address Type 2 Diabetes Self-Management: A Randomized Pilot Trial. Sci Diabetes Self Manag Care. 2022;48(6):492-504.

Coombes JS, Keating SE, Mielke GI, et al. Personal Activity Intelligence e-Health Program in People with Type 2 Diabetes: A Pilot Randomized Controlled Trial. Med Sci Sports Exerc. 2022;54(1):18-27.

DiNardo MM, Greco C, Phares AD, et al. Effects of an integrated mindfulness intervention for veterans with diabetes distress: a randomized controlled trial. BMJ Open Diabetes Res Care. 2022;10(2):e002631.

Kim Y, Lee H, Seo JM. Integrated Diabetes Self-Management Program Using Smartphone Application: A Randomized Controlled Trial. West J Nurs Res. 2022;44(4):383-394.

Riangkam, C., Sriyuktasuth, A., Pongthavornkamol, K., Kusakunniran, W., & Sriwijitkamol, A. (2022). Effects of a mobile health diabetes self-management program on HbA1C, self-management and patient satisfaction in adults with uncontrolled type 2 diabetes: a randomized controlled trial. Journal of Health Research, 36(5), 878-888.

Ruissen MM, Torres-Peña JD, Uitbeijerse BS, et al. Clinical impact of an integrated e-health system for diabetes self-management support and shared decision making (POWER2DM): a randomised controlled trial. Diabetologia. 2023;66(12):2213-2225.

- *App inaccessible to patients*

Feng Y, Zhao Y, Mao L, et al. The Effectiveness of an eHealth Family-Based Intervention Program in Patients With Uncontrolled Type 2 Diabetes Mellitus (T2DM) in the Community Via WeChat: Randomized Controlled Trial. JMIR Mhealth Uhealth. 2023;11:e40420.

Luo E, Wan J, Su M, et al. Efficacy of a basal insulin dose management smartphone application for controlling fasting blood glucose in patients with type-2 diabetes mellitus: A single-centre, randomised clinical study. Clin Endocrinol (Oxf). 2023;99(4):361-369.

Poonprapai P, Lerkiatbundit S, Saengcharoen W. Family support-based intervention using a mobile application provided by pharmacists for older adults with diabetes to improve glycaemic control: a randomised controlled trial. Int J Clin Pharm. 2022;44(3):680-688.

***Ineligible participants***

Gonzalez-Sanchez J, Recio-Rodriguez JI, Fernandez-delRio A, et al. Using a smartphone app in changing cardiovascular risk factors: A randomized controlled trial (EVIDENT II study). Int J Med Inform. 2019;125:13-21.

Li A, Del Olmo MG, Fong M, et al. Effect of a smartphone application (Perx) on medication adherence and clinical outcomes: a 12-month randomised controlled trial. BMJ Open. 2021;11(8):e047041.

Oh SW, Kim KK, Kim SS, Park SK, Park S. Effect of an Integrative Mobile Health Intervention in Patients With Hypertension and Diabetes: Crossover Study. JMIR Mhealth Uhealth. 2022;10(1):e27192.

Wong AKC, Bayuo J, Wong FKY, Chow KKS, Wong SM, Lau ACK. The Synergistic Effect of Nurse Proactive Phone Calls With an mHealth App Program on Sustaining App Usage: 3-Arm Randomized Controlled Trial. J Med Internet Res. 2023;25:e43678.

***Ineligible outcomes***

Adepoju OE, Bolin JN, Ohsfeldt RL, et al. Can chronic disease management programs for patients with type 2 diabetes reduce productivity-related indirect costs of the disease? Evidence from a randomized controlled trial. Popul Health Manag. 2014;17(2):112-120.

Adepoju OE, Bolin JN, Phillips CD, et al. Effects of diabetes self-management programs on time-to-hospitalization among patients with type 2 diabetes: a survival analysis model. Patient Educ Couns. 2014;95(1):111-117.

Alanzi T, Bah S, Alzahrani S, Alshammari S, Almunsef F. Evaluation of a mobile social networking application for improving diabetes Type 2 knowledge: an intervention study using WhatsApp. J Comp Eff Res. 2018;7(9):891-899.

Bailey DP, Mugridge LH, Dong F, Zhang X, Chater AM. Randomised Controlled Feasibility Study of the MyHealthAvatar-Diabetes Smartphone App for Reducing Prolonged Sitting Time in Type 2 Diabetes Mellitus. Int J Environ Res Public Health. 2020;17(12):4414.

Bloss CS, Wineinger NE, Peters M, et al. A prospective randomized trial examining health care utilization in individuals using multiple smartphone-enabled biosensors. PeerJ. 2016;4:e1554.

Castelnuovo G, Manzoni GM, Cuzziol P, et al. TECNOB Study: Ad Interim Results of a Randomized Controlled Trial of a Multidisciplinary Telecare Intervention for Obese Patients with Type-2 Diabetes. Clin Pract Epidemiol Ment Health. 2011;7:44-50.

Chao DY, Lin TM, Ma WY. Enhanced Self-Efficacy and Behavioral Changes Among Patients With Diabetes: Cloud-Based Mobile Health Platform and Mobile App Service. JMIR Diabetes. 2019;4(2):e11017.

Choi JS, Ma D, Wolfson JA, Wyman JF, Adam TJ, Fu HN. Associations Between Psychosocial Needs, Carbohydrate-Counting Behavior, and App Satisfaction: A Randomized Crossover App Trial on 92 Adults With Diabetes. Comput Inform Nurs. 2023;41(12):1026-1036.

Choudhry NK, Isaac T, Lauffenburger JC, et al. Effect of a Remotely Delivered Tailored Multicomponent Approach to Enhance Medication Taking for Patients With Hyperlipidemia, Hypertension, and Diabetes: The STIC2IT Cluster Randomized Clinical Trial. JAMA Intern Med. 2018;178(9):1182-1189.

Dincer B, Bahçecik N. The effect of a mobile application on the foot care of individuals with type 2 diabetes: a randomized controlled study. Health Education Journal. 2020;80(4): 425-437.

Firdaus MKZH, Jittanoon P, Boonyasopun U, Che Hasan MK. The effect of mHealth program on behavior modification and health outcomes among patients with diabetes: A randomized controlled trial study. Belitung Nurs J. 2023;9(5):437-447.

Fu HN, Adam TJ, Konstan JA, Wolfson JA, Clancy TR, Wyman JF. Influence of Patient Characteristics and Psychological Needs on Diabetes Mobile App Usability in Adults With Type 1 or Type 2 Diabetes: Crossover Randomized Trial. JMIR Diabetes. 2019;4(2):e11462.

Garner SL, Young P, Fendt M, et al. Effectiveness of a Culturally Responsive mHealth Gaming Application to Improve Diabetes Health Literacy in India: A Randomized Controlled Trial. Comput Inform Nurs. 2023;41(10):796-804.

Höchsmann C, Infanger D, Klenk C, Königstein K, Walz SP, Schmidt-Trucksäss A. Effectiveness of a Behavior Change Technique-Based Smartphone Game to Improve Intrinsic Motivation and Physical Activity Adherence in Patients With Type 2 Diabetes: Randomized Controlled Trial. JMIR Serious Games. 2019;7(1):e11444.

Holmen H, Wahl A, Torbjørnsen A, Jenum AK, Småstuen MC, Ribu L. Stages of change for physical activity and dietary habits in persons with type 2 diabetes included in a mobile health intervention: the Norwegian study in RENEWING HEALTH. BMJ Open Diabetes Res Care. 2016;4(1):e000193.

Hummel M, Bonn SE, Trolle Lagerros Y. The effect of the smartphone app DiaCert on health related quality of life in patients with type 2 diabetes: results from a randomized controlled trial. Diabetol Metab Syndr. 2022;14(1):192.

Iversen MM, Igland J, Smith-Strøm H, et al. Effect of a telemedicine intervention for diabetes-related foot ulcers on health, well-being and quality of life: secondary outcomes from a cluster randomized controlled trial (DiaFOTo). BMC Endocr Disord. 2020;20(1):157.

Jiwani R, Wang J, Berndt A, et al. Changes in Patient-Reported Outcome Measures With a Technology-Supported Behavioral Lifestyle Intervention Among Patients With Type 2 Diabetes: Pilot Randomized Controlled Clinical Trial. JMIR Diabetes. 2020;5(3):e19268.

Kilic M, Karadağ A. Developing and Evaluating a Mobile Foot Care Application for Persons With Diabetes Mellitus: A Randomized Pilot Study. Wound Manag Prev. 2020;66(10):29-40.

Lee MK, Lee DY, Ahn HY, Park CY. A Novel User Utility Score for Diabetes Management Using Tailored Mobile Coaching: Secondary Analysis of a Randomized Controlled Trial. JMIR Mhealth Uhealth. 2021;9(2):e17573.

Mahoney E, Glezer S, Baccari L, Lebowitz J, Yue W, Klonoff D. Use of a diabetes self-management application in combination with a 4 mm pen needle and its impact on glycemic variability and patient-reported outcomes in people with type 2 diabetes using basal-bolus insulin therapy. Clinical Diabetology. 2022;11(3), 156-164.

Marques ADB, Moreira TMM, Mourão LF, et al. Mobile Application for Adhering to Diabetic Foot Self-care: Randomized Controlled Clinical Trial. Comput Inform Nurs. 2023;41(11):877-883.

Mayberry LS, Berg CA, Greevy RA, et al. Mixed-Methods Randomized Evaluation of FAMS: A Mobile Phone-Delivered Intervention to Improve Family/Friend Involvement in Adults' Type 2 Diabetes Self-Care. Ann Behav Med. 2021;55(2):165-178.

Sittig S, Wang J, Iyengar S, Myneni S, Franklin A. Incorporating Behavioral Trigger Messages Into a Mobile Health App for Chronic Disease Management: Randomized Clinical Feasibility Trial in Diabetes. JMIR Mhealth Uhealth. 2020;8(3):e15927.

Sunil Kumar D, Prakash B, Subhash Chandra BJ, Kadkol PS, Arun V, Thomas JJ. An android smartphone-based randomized intervention improves the quality of life in patients with type 2 diabetes in Mysore, Karnataka, India. Diabetes Metab Syndr. 2020;14(5):1327-1332.

Thorsen IK, Yang Y, Valentiner LS, et al. The Effects of a Lifestyle Intervention Supported by the InterWalk Smartphone App on Increasing Physical Activity Among Persons With Type 2 Diabetes: Parallel-Group, Randomized Trial. JMIR Mhealth Uhealth. 2022;10(9):e30602.

Torbjørnsen A, Småstuen MC, Jenum AK, Årsand E, Ribu L. Acceptability of an mHealth App Intervention for Persons With Type 2 Diabetes and its Associations With Initial Self-Management: Randomized Controlled Trial. JMIR Mhealth Uhealth. 2018;6(5):e125.

Unsworth R, Armiger R, Jugnee N, et al. Safety and Efficacy of an Adaptive Bolus Calculator for Type 1 Diabetes: A Randomized Controlled Crossover Study. Diabetes Technol Ther. 2023;25(6):414-425.

Wungrath J, Autorn N. Effectiveness of Line application and telephone-based counseling to improve medication adherence: A randomized control trial study among uncontrolled type 2 diabetes patients. Health Promot Perspect. 2021;11(4):438-443.

Young HM, Miyamoto S, Dharmar M, Tang-Feldman Y. Nurse Coaching and Mobile Health Compared With Usual Care to Improve Diabetes Self-Efficacy for Persons With Type 2 Diabetes: Randomized Controlled Trial. JMIR Mhealth Uhealth. 2020;8(3):e16665.

***Ineligible study design***

Adu MD, Malabu UH, Malau-Aduli AEO, Drovandi A, Malau-Aduli BS. Efficacy and Acceptability of My Care Hub Mobile App to Support Self-Management in Australians with Type 1 or Type 2 Diabetes. Int J Environ Res Public Health. 2020;17(7):2573.

Bonn SE, Alexandrou C, Hjörleifsdottir Steiner K, et al. App-technology to increase physical activity among patients with diabetes type 2 - the DiaCert-study, a randomized controlled trial. BMC Public Health. 2018;18(1):119.

Brož J. Clinical efficacy of a smartphone-based integrated online real-time diabetes care system in Type 2 diabetes patients. Intern Med J. 2021;51(3):464.

Burnside M, Lewis D, Crocket H, et al. CREATE (Community deRivEd AutomaTEd insulin delivery) trial. Randomised parallel arm open label clinical trial comparing automated insulin delivery using a mobile controller (AnyDANA-loop) with an open-source algorithm with sensor augmented pump therapy in type 1 diabetes. J Diabetes Metab Disord. 2020;19(2):1615-1629.

Canonico ME, Hsia J, Guthrie NL, et al. Cognitive behavioral therapy delivered via digital mobile application for the treatment of type 2 diabetes: Rationale, design, and baseline characteristics of a randomized, controlled trial. Clin Cardiol. 2022;45(8):850-856.

Chi CJ, Yu YC, Du YF, et al. Comparing a Social and Communication App, Telephone Intervention, and Usual Care for Diabetes Self-Management: 3-Arm Quasiexperimental Evaluation Study. JMIR Mhealth Uhealth. 2020;8(6):e14024.

Doddaiah SK, Prakash B, Subhash Chandra BJ, et al. Effectiveness of smartphone-based intervention on the perceptions of type 2 Diabetes Mellitus patients in Mysuru, Karnataka, India. Obesity Medicine. 2020;20, 100295.

Farooqi MH, Abdelmannan DK, Al Buflasa MM, et al. The Impact of Telemonitoring on Improving Glycemic and Metabolic Control in Previously Lost-to-Follow-Up Patients with Type 2 Diabetes Mellitus: A Single-Center Interventional Study in the United Arab Emirates. Int J Clin Pract. 2022;2022:6286574.

Feuerstein-Simon C, Bzdick S, Padmanabhuni A, Bains P, Roe C, Weinstock RS. Use of a Smartphone Application to Reduce Hypoglycemia in Type 1 Diabetes: A Pilot Study. J Diabetes Sci Technol. 2018;12(6):1192-1199.

Hasanah N, Ikawati Z, Zainal ZA. The effectiveness of smartphone application-based education “teman diabetes” on clinical outcomes of type-2 diabetes mellitus patients. Research Journal of Pharmacy and Technology. 2021;14(7), 3625-3630.

Hooshmandja M, Mohammadi A, Esteghamti A, Aliabadi K, Nili M. Effect of mobile learning (application) on self-care behaviors and blood glucose of type 2 diabetic patients. J Diabetes Metab Disord. 2019;18(2):307-313.

Kannan S, Shivaprasad KS, Khadilkar K, Rajesh S, Sigamani A, Annapandian VM. Achieving Higher Levels of Efficiency in a Diabetes Outpatient Clinic With Improved Patient Care Quality Through the Use of Digital Clinical Assistant Software Application. J Diabetes Sci Technol. 2020;14(2):496-498.

Lee EY, Yun JS, Cha SA, et al. Personalized Type 2 Diabetes Management Using a Mobile Application Integrated with Electronic Medical Records: An Ongoing Randomized Controlled Trial. Int J Environ Res Public Health. 2021;18(10):5300. Published 2021 May 16. doi:10.3390/ijerph18105300

Maxwell TK, Charmant CO, Volkening LK, Laffel LM, Katz ML. A Randomized Pilot Trial Using Mobile Health and Financial Incentives to Motivate Heart-Healthy Behaviors in Adolescents With Type 1 Diabetes. J Diabetes Sci Technol. 2023;17(3):855-856.

Merwin RM, Moskovich AA, Babyak M, et al. An open trial of app-assisted acceptance and commitment therapy (iACT) for eating disorders in type 1 diabetes. J Eat Disord. 2021;9(1):6.

Osborn CY, van Ginkel JR, Marrero DG, Rodbard D, Huddleston B, Dachis J. One Drop | Mobile on iPhone and Apple Watch: An Evaluation of HbA1c Improvement Associated With Tracking Self-Care. JMIR Mhealth Uhealth. 2017;5(11):e179.

de Oliveira FM, Calliari LEP, Feder CKR, et al. Efficacy of a glucose meter connected to a mobile app on glycemic control and adherence to self-care tasks in patients with T1DM and LADA: a parallel-group, open-label, clinical treatment trial. Arch Endocrinol Metab. 2021;65(2):185-197.

Park SW, Kim G, Hwang YC, Lee WJ, Park H, Kim JH. Validation of the effectiveness of a digital integrated healthcare platform utilizing an AI-based dietary management solution and a real-time continuous glucose monitoring system for diabetes management: a randomized controlled trial. BMC Med Inform Decis Mak. 2020;20(1):156.

***Inaccessible publication***

Felker GM, Sharma A, Mentz RJ, et al. A Randomized Controlled Trial of Mobile Health Intervention in Patients With Heart Failure and Diabetes. J Card Fail. 2022;28(11):1575-1583.

Pamungkas RA, Usman AM, Chamroonsawasdi K, Abdurrasyid. A smartphone application of diabetes coaching intervention to prevent the onset of complications and to improve diabetes self-management: A randomized control trial. Diabetes Metab Syndr. 2022;16(7):102537.

Sachmechi I, Salam S, Amini M, Khan R, Spitznogle A, Belen T. Frequent Monitoring of Blood Glucose Levels via a Remote Patient Monitoring System Helps Improve Glycemic Control. Endocr Pract. 2023;29(6):441-447.

Tan NC, Tyagi S, Lee CS, et al. Effectiveness of an algorithm-driven home telemonitoring system on the metabolic control and self-care behaviour of Asian adults with type-2 diabetes mellitus: A randomised controlled trial. J Telemed Telecare. 2023;0(0).

Yang L, Xu J, Kang C, et al. Effects of Mobile Phone-Based Telemedicine Management in Patients With Type 2 Diabetes Mellitus: A Randomized Clinical Trial. Am J Med Sci. 2022;363(3):224-231.

# **Supplementary Table 5 – Study and sample characteristics of included studies**

| **Author, year, country** | **Study design, duration** | **Overall participants** | **Delivery mode** | **Theoretical basis** | **User involvement** | **Technology automation** | **Additional HCP involvement** | **Target behaviours** | **Impact on glycaemic control**  **expressed as**  **MD (SD)** |
| --- | --- | --- | --- | --- | --- | --- | --- | --- | --- |
| Agarwal, 2019, Canada | RCT,  3 m | n=223 adults with type 2 diabetes | - Study device | - Transtheoretical Model of Behaviour Change | - | - Customized, evidence-based messages |  | - Healthy eating - Being active - Monitoring | I: -0·67 (1·70)  C: -0·62 (1·63) |
| Alanzi, 2018, Saudi Arabia | RCT,  6 m | n=20 adults up to 50 years of age with type 2 diabetes | - Study device |  | - Preliminary mixed-methods design - Usability testing |  | - Videos and additional communication with HCPs | - Healthy coping - Monitoring | I: -0·60 (0·31)  C: -0·05 (0·35) |
| Alfonsi, 2020, Canada | Pilot RCT,  3 m | n=44 children aged 10-17 with type 1 diabetes | - Personal device |  | - Co-design with intended users (dietitians, diabetes educators, patients) - Usability testing |  |  | - Health eating - Monitoring | I: -0·35 (1·51)  C: 0·45 (1·33) |
| Anzaldo Campos, 2016, Mexico | RCT,  10 m | n=201 adults aged 18-75 with type 2 diabetes | - Study device |  |  |  | - Care management by multidisciplinary team - Peer-led group education component | - Healthy eating - Being active - Taking medication - Monitoring | I: -3·02 (2·83)  C: -2·63 (3·73) |
| Baron, 2017, United Kingdom | RCT,  9 m | n=81 adults with either type 1 or type 2 diabetes | - Study device | - Social cognitive theory - Model of illness beliefs - Technology Acceptance Model | - Co-design with HCP advice and user feedback |  | - Support by MTH nurse (lifestyle education, feedback on clinical readings) | - Being active - Taking medication - Monitoring | I: -0·51 (1·50)  C: 0·05 (1·47) |
| Bee, 2016, Singapore | Pilot RCT, 6 m | n=66 individuals with type 2 diabetes | - Personal device |  |  | - Suggested insulin doses | - Possibility to issue readings to endocrinologists | - Monitoring - Reducing risk | I: -1·59 (1·62)  C: -1·48 (0·97) |
| Boels, 2019, The Netherlands | RCT,  6 m | n=230 adults aged 40-70 with type 2 diabetes | - Personal device | - Health Belief Model - Transtheoretical Model of BC - Fogg Behaviour Model | - Co-design with dietitian, physiotherapist and nurse |  |  | - Healthy eating - Being active - Monitoring - Reducing risk | I: -0·10 (1·30)  C: -0·10 (1.21) |
| Castensøe-Seidenfaden, 2018, Denmark | RCT,  12 m | n=151 young people aged 14-22 with type 1 diabetes | - Personal device |  | - Co-design with patients, parents and HCPs |  | - Message exchange with HCPs | - Healthy eating | I: 0·00 (1·48)  C: -0·20 (1·18) |
| Charpentier, 2011, France | RCT,  6 m | n=121 adults with type 1 diabetes | - Study device |  |  | - Automatic adjustment of carbohydrate ratio |  | - Healthy eating - Monitoring - Reducing risk | I: -0·49 (0·89)  C: 0·18 (0·83) |
| Chatzakis, 2019, Greece | RCT,  12 m | n=80 children aged 7-17 with type 1 diabetes | - Personal device |  |  | - Automatic calculation of required bolus insulin dose |  | - Healthy eating - Being active - Monitoring - Reducing risk | I: -1·05 (0·77)  C: -0·1 (0·59) |
| Christensen, 2022, Denmark | RCT,  6 m | n=170 adults aged 18-70 with type 2 diabetes | - Personal device |  | - Qualitative interview studies with patients, GPs, ehealth coaches |  | - Support by health coaches | - Healthy eating - Being active - Monitoring - Reducing risk - Problem solving | I: -0·76 (1·15)  C: -0·61 (0·72) |
| Derkaoui, 2023, Morocco | RCT,  3 m | n=62 patients with type 1 diabetes | - Personal device |  | - Co-design with medical doctors | - Automatic suggestion of insulin dose to be injected |  | - Healthy eating - Being active - Monitoring | I: -0·90 (1·70)  C: -0·20 (0·90) |
| Drion, 2015, The Netherlands | RCT,  3 m | n=63 adults with type 1 diabetes | - Personal device |  |  |  | - Direct communication with diabetes nurse | - Healthy eating - Being active - Monitoring | - |
| Forjouh, 2014, United States of America | RCT,  12 m | n=376 adults with type 2 diabetes | - Study device |  |  |  |  | - Healthy eating - Being active - Taking medication - Monitoring | - |
| Franc, 2020, France | RCT,  12 m | n=434 individuals with either type 1 or type 2 diabetes | - Personal device |  |  | - Automatic adjustment of insulin dose |  | - Healthy eating - Being active - Monitoring - Reducing risk | - |
| Frias, 2017, United States of America | Pilot cluster RCT,  3 m | n=109 individuals with type 2 diabetes | - Personal device |  |  |  | - Provider support in timely and targeted therapy optimization | - Being active - Taking medication - Monitoring | I: -0·08 (1·39)  C: 0·26 (1·88) |
| Gong, 2020, Australia | RCT,  12 m | n=187 adults with type 2 diabetes | - Personal device | - Transtheoretical model - Social cognitive theory | - Co-design with Bupa Foundation and technology company | - Conversational agent that guides individual progress | - Support by program coordinator to maintain discussion forum and foster program use | - Healthy eating - Being active - Taking medication - Monitoring - Problem solving | I: -0·33 (1·43)  C: -0·28 (1·41) |
| Goyal, 2017, Canada | RCT,  12 m | n=92 adolescents aged 11-16 with type 1 diabetes | - Study device |  | - Qualitative ethnographic interviews - Focus group session | - Algorithm that detects OOR readings and autonomously identifies possible causes |  | - Monitoring - Reducing risk - Problem solving | I: 0·00 (1·04)  C: 0·04 (0·97) |
| Grady, 2017, United Kingdom | RCT,  6 m | n= 128 adults aged 16-70 with either type 1 or type 2 diabetes | - Study device |  |  |  | - Support by HCP via text messages with diabetes-related advice | - Monitoring - Reducing risk | I: -0·66 (0·94)  C: -0·56 (0·87) |
| Gunawardena, 2019, Sri Lanka | Pilot RCT,  6 m | n=67 adults aged 18-80 with type 2 diabetes | - Personal device |  |  | - Automatic calculation of bolus insulin dose needed |  | - Healthy eating - Being active - Taking medication - Monitoring | I: -2·32 (0·89)  C: -1·27 (1·10) |
| Heald, 2023, United Kingdom | RCT,  6 m | n=197 adults with type 2 diabetes | - Personal device |  |  |  | - Support by nurses | - Monitoring - Problem solving | I: -0·60 (1·33)  C: 0·00 (1·30) |
| Hermanns, 2023, Germany | RCT,  3 m | n=251 adults with type 2 diabetes | - Personal device |  |  | - Automatic recommendation of basal insulin doses | - Support by physicians with use of portal for participant management | - Monitoring | I: -0·98 (0. ·89)  C: -0·37 (0·96) |
| Hsia, 2022, United States of America | RCT,  6 m | n=669 adults aged 18-75 with type 2 diabetes | - Personal device |  | - Human-centred software design |  |  | - Healthy coping - Healthy eating - Being active - Monitoring - Reducing risk | I: -0·28 (1·20)  C: 0·11 (1·18) |
| Hilmarsdóttir, 2020, Iceland | RCT,  6 m | n=30 adults aged 18-75 with type 2 diabetes | - Personal device |  |  |  |  | - Healthy coping - Healthy eating - Being active - Taking medication - Monitoring | I: -0·70 (1·61)  C: -0·10 (1·54) |
| Höchsmann, 2019, Switzerland | RCT,  6 m | n=36 adults aged 45-70 with type 2 diabetes | - Personal device | - Self-determination theory | - Development phase with 4 user studies | - Automatic selection of intensity progression for exercise regimens |  | - Being active - Monitoring | I: 0·00 (0·59)  C: 0·10 (0·81) |
| Holmen – Torbjorsen, 2014, Norway | RCT,  12 m | n=151 adults with type 2 diabetes | - Study device |  | - Co-design with patients with diabetes |  |  | - Health eating - Being active - Monitoring - Reducing risk | I: -0·31 (1·04)  C: -0·16 (1·25) |
| Huang, 2019, Singapore | Pilot RCT,  3 m | n=41 adults 21+ with type 2 diabetes | - Personal device |  |  |  |  | - Taking medication - Monitoring | I: 0·30 (1·93)  C: 0·80 (1·92) |
| Jiang, 2022, Singapore | RCT,  6 m | n=114 adults 21+ with type 2 diabetes | - Personal device | - Self-efficacy theory | - Usability testing with experts, HCPs and patients |  | - Support by nurses with verbal persuasion and reassurance | - Healthy eating - Being active - Monitoring | I: -1·63 (1·61)  C: -1·23 (1·64) |
| Karhula, 2015, Finland | RCT,  12 m | n=250 individuals with type 2 diabetes | - Study device |  |  |  | - Regular calls by health coaches to provide support | - Being active - Monitoring | I: 0·04 (0·80)  C: 0·18 (0·76) |
| Katz, 2022, United States of America | RCT,  3 m | n=120 adults aged 18-70 with type 1 or type 2 diabetes | - Personal device - Study device |  |  |  | - Support by HCPs in preparing a 14-day web-based report | - Monitoring - Reducing risk | I: -0·99 (1·20)  C: -0·63 (1·20) |
| Kim, 2019, South Korea | RCT,  6 m | n=172 adults aged 19-80 with type 2 diabetes | - Personal device |  |  | - Insulin dosing algorithm | - Remote adjustment of the insulin titration algorithm by HCPs | - Healthy eating - Being active - Monitoring - Reducing risk | I: -0·40 (0·68)  C: 0·10 (0·74) |
| Kirwan, 2013, Australia | RCT,  9 m | n=72 adults aged 18-65 with type 1 diabetes | - Personal device |  |  |  | - Weekly personalised messages by CDEs | - Healthy eating - Being active - Taking medication - Monitoring - Reducing risk | I: -1·28 (0·94)  C: 0·07 (0·94) |
| Kleinman, 2017, India | RCT,  6 m | n=61 adults aged 18-65 with type 2 diabetes | - Personal device | - Health belief model - Health action process approach - Theory of planned behaviour - Self-efficacy theory |  |  | - Response to system-generated alerts by health coaches | - Taking medication - Monitoring | I: -1·50 (1·10)  C: -0·80 (1·60) |
| Kusnanto, 2019, Indonesia | RCT,  3 m | n=30 adults with type 2 diabetes | - Personal device |  |  |  |  | - Healthy eating - Being active - Monitoring | I: -1·10 (1·18)  C: -0·27 (0·86) |
| Lee, 2018, South Korea | RCT,  12 m | n=148 adults aged 19+ with type 2 diabetes | - Personal device |  |  |  | - Bidirectional communication through the app with clinicians | - Healthy eating - Being active - Taking medication - Monitoring - Reducing risk | I: -0·60 (1·22)  C: -0·10 (1·24) |
| Lee, 2022, South Korea | RCT,  6 m | n=269 adults aged 19-74 with type 2 diabetes | - Personal device |  |  | - Automated messages based on individual lifestyle questionnaires |  | - Healthy eating - Being active - Taking medication - Monitoring - Reducing risk | I: -0·80 (1·70)  C: -0·60 (1·10) |
| Lee, 2023, South Korea | RCT,  6 m | n=294 adults with type 2 diabetes | - Personal device |  |  |  |  | - Healthy eating - Being active - Monitoring | I: -0·28 (0·53)  C: -0·13 (0·73) |
| Leong, 2022, Taiwan | RCT,  3 m | n=120 adults aged 20+ with type 2 diabetes | - Personal device |  | - Co-design with HCPs and students |  | - Communication with HCPs through the messaging feature of the app | - Healthy eating - Taking medication - Reducing risk | I: 0·07 (0·70)  C: 0·01 (0·70) |
| Li, 2021, China | RCT,  3 m | n=101 adults aged 18-64 with type 2 diabetes | - Personal device - Study device |  |  |  |  | - Being active - Monitoring | I: -0·72 (1·09)  C: -0·57 (1·35) |
| Lim, 2021, Singapore | RCT,  6 m | n=204 adults aged 21-75 with type 2 diabetes | - Personal device | - Obesity-Related Behavioural Intervention Trials (ORBIT) Model |  | - Automatic generation of healthier food alternatives | - Support by local dietitians | - Healthy coping - Healthy eating - Being active - Monitoring - Reducing risk - Problem solving | I: -0·70 (1·20)  C: -0·30 (1·00) |
| Quinn, 2011, United States of America | Cluster RCT,  6 m | n=79 adults aged 18-64 with type 2 diabetes | - Study device |  | - Co-design with endocrinologists and CDEs | - Automated, real-time messages based on inputted data | - Support to complement automated messages by CDEs | - Healthy eating - Taking medication - Monitoring - Reducing risk | I: -1·60 (1·44)  C: -0·70 (1·57) |
| Rossi, 2013, Italy | RCT,  6 m | n=127 adults with type 1 diabetes | - Personal device |  |  | - Automatic calculation of appropriate insulin dose | - Regular feedback on collected data by HCPs | - Healthy eating - Being active - Monitoring - Reducing risk | I: -0·49 (0·71)  C: -0·48 (0·72) |
| Skrøvseth, 2015, Norway | RCT,  3 m | n=30 adults with type 1 diabetes | - Personal device |  | - Co-design with users | - Data-driven feedback based on patient data |  | - Healthy eating - Being active - Monitoring - Reducing risk | I: -0·63 (0·70)  C: -0·57 (0·95) |
| Sun, 2019, China | RCT,  6 m | n=91 adults 65+ with type 2 diabetes | - Personal device |  |  |  | - Advice and personalized dietary recommendations by medical team | - Healthy eating - Being active - Monitoring - Reducing risk | I: -1·07 (0·89)  C: -0·62 (1·00) |
| Sunil Kumar, 2021, India | RCT,  6 m | n=300 adults aged 18-65 with type 2 diabetes | - Personal device |  |  | - Automatic suggestion of necessary caloric intake |  | - Healthy eating - Taking medication - Monitoring | I: -0·26 (0·90)  C: 0·13 (1·21) |
| Timurtas, 2021, Turkey | RCT,  3 m | n=75 adults aged 30-65 with type 2 diabetes | - Personal device | - Transtheoretical model | - Delphi method with experts on type 2 diabetes |  | - Communication with physiotherapists through the message feature of the app | - Being active - Monitoring | I: -0·60 (0·36)  C: -0·90 (0·90) |
| Wang, 2018, United States of America | Pilot RCT,  6 m | n=26 adults aged 21-75 with type 2 diabetes | - Study device | - Self-regulation theory - Social learning theory | - Focus groups to assess intervention acceptability |  | - Group session at community health centres | - Healthy eating - Being active - Taking medication - Monitoring - Reducing risk - Problem solving | I: -1·50 (1·88)  C: 0·00 (1·93) |
| Wang, 2019, China | RCT,  6 m | n=120 adults aged 30-60 with type 2 diabetes | - Personal device |  |  | - Automatic suggestions based on BG control | - One-to-one interaction with physicians - Timely follow-up by diabetes nurse | - Healthy eating - Being active - Taking medication - Monitoring | I: -1·50 (1·96)  C: -0·76 (1·97) |
| Wang, 2020, Singapore | Pilot RCT,  3 m | n=40 adults aged 21+ with type 2 diabetes | - Personal device | - Self-efficacy theory | - Usability testing by HCPs and patients |  | - Support through web portal by research nurse | - Healthy eating - Taking medication - Monitoring | I: -1·47 (1·65)  C: -0·66 (1·58) |
| Wayne, 2015, Canada | RCT,  6 m | n=97 adults up to 70 years of age with type 2 diabetes | - Study device |  | - Semi-structured interviews to refine user interface |  | - Control of participants’ inputs by health coaches | - Healthy coping - Healthy eating - Being active - Monitoring | I: -0·81 (1·22)  C: -0·76 (1·22) |
| Xu H, 2021, China | RCT,  6 m | n=89 adults aged 18-65 with type 2 diabetes | - Personal device |  |  | - Individualized self-management programs based on the results | - Online consultation with nurses | - Healthy eating - Being active - Taking medication - Monitoring | I: -1·35 (1·67)  C: -0·82 (1·47) |
| Xu Y, 2021, China | RCT,  6 m | n=60 young adults aged 10-19 with type 1 diabetes | - Study device |  | - Design adjustment based on a national survey to patients with diabetes |  | - Real-time interaction with doctors on the platform | - Healthy eating - Being active - Monitoring - Reducing risk | I: -0·52 (1·00)  C: -0·23 (1·85) |
| Yang, 2021 | RCT,  12 m | n=100 adults aged 40-60 with type 2 diabetes | - Personal device |  |  | - Automatic warnings based on glycaemic intake | - Monthly phone visit by clinicians | - Healthy eating - Taking medication - Monitoring | I: -1·26 (1·70)  C: -0·01 (1·92) |
| Yu, 2019, China | RCT,  6 m | n=185 adults aged 35-65 with type 2 diabetes | - Personal device |  |  |  | - Real-time communication with certified clinicians | - Healthy eating - Being active - Monitoring - Reducing risk | I: -1·10 (0·30)  C: -1·10 (0·40) |
| Zhai, 2020, China | RCT,  6 m | n=118 adults aged 18-60 with type 2 diabetes | - Personal device |  |  |  | - Online daily instruction by education nurses | - Healthy coping - Healthy eating - Being active - Taking medication - Monitoring - Reducing risk | I: -2·15 (0·99)  C: -1·83 (1·02) |
| Zhang, 2019, China | RCT,  6 m | n=234 adults aged 18-65 with either type 1 or type 2 diabetes | - Personal device |  |  |  |  | - Healthy eating - Being active - Taking medication - Monitoring - Reducing risk | I: -1·37 (1·48)  C: -1·34 (1·50) |
| Zhou, 2016, China | RCT,  3 m | n=100 adults aged 18-74 with either type 1 or type 2 diabetes | - Personal device |  |  |  | - Advice and feedbacks by virtual endocrinologists | - Healthy eating - Being active - Taking medication - Monitoring - Reducing risk | I: -1·95 (1·91)  C: -0·79 (2·09) |

**Legend**: BC = behaviour change; BG = blood glucose; CDE = certified diabetes educator; GP = general practitioner; HCP = healthcare professional; MD = mean difference; OOR = out-of-range; RCT = randomised controlled trial; SD = standard deviation.

# **Supplementary Figure 6 – Prevalence of Behaviour Change Techniques in selected app-based DBCIs**

**
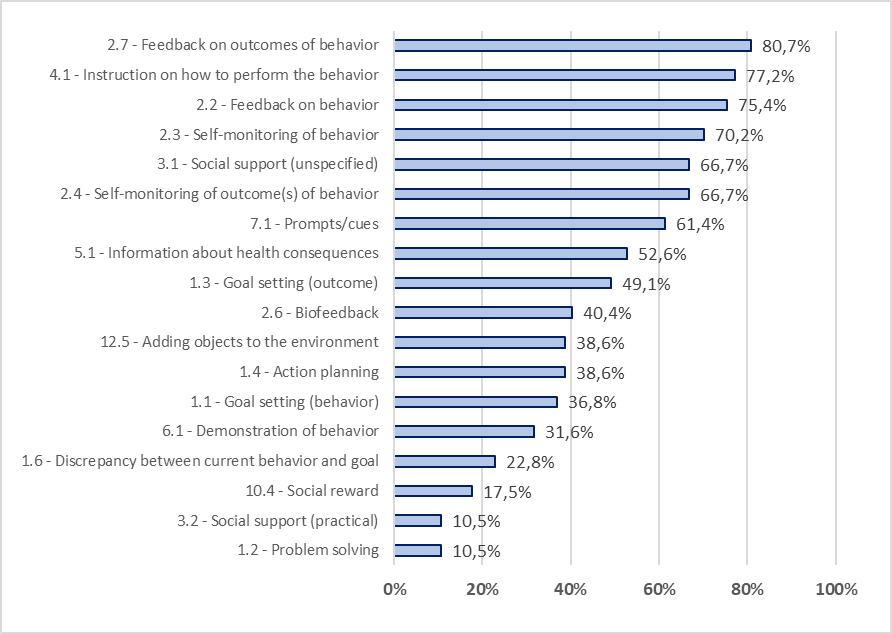
**

**Legend*:*** Additional BCTs appearing in less than 10% of the selected studies included: 1.5 – review behavior goals (9%); 3.3 – social support (emotional) (9%); 9.1 – credible source (7%); 6.2 – social comparison (5%); 7.3 – reduce prompts/cues (5%); 8.7 – graded tasks (5%); 1.7 - review outcome goal(s) (4%); 8.1 - behavioral practice / rehearsal (4%); 2.1 - monitoring of behavior by others without feedback (2%); 2.5 - monitoring of outcome(s) of behavior without feedback (2%); 10.2 – material reward (behavior) (2%); 10.3 – non-specific reward (2%); 10.5 – social incentive (2%); 10.10 – reward (outcome) (2%); 10.11 – future punishment (2%).

# **Supplementary Figure 7 – Risk of Bias for HbA1c changes: summary plot for individually-randomized studies (N=55)**

**
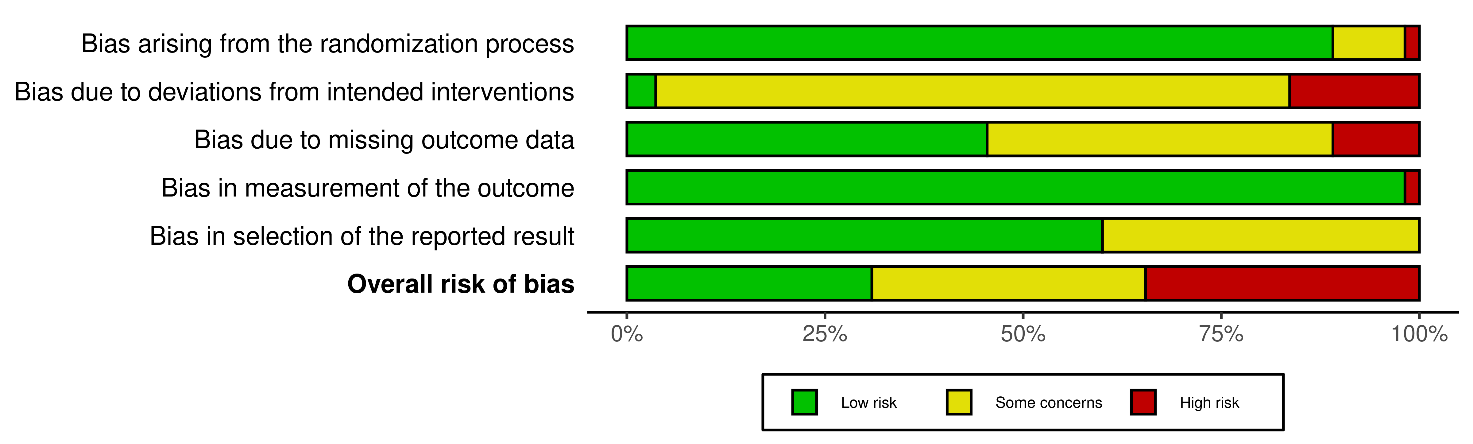
**

# **Supplementary Figure 8 – Study-level details of the risk of bias assessment for individually-randomized studies**

**
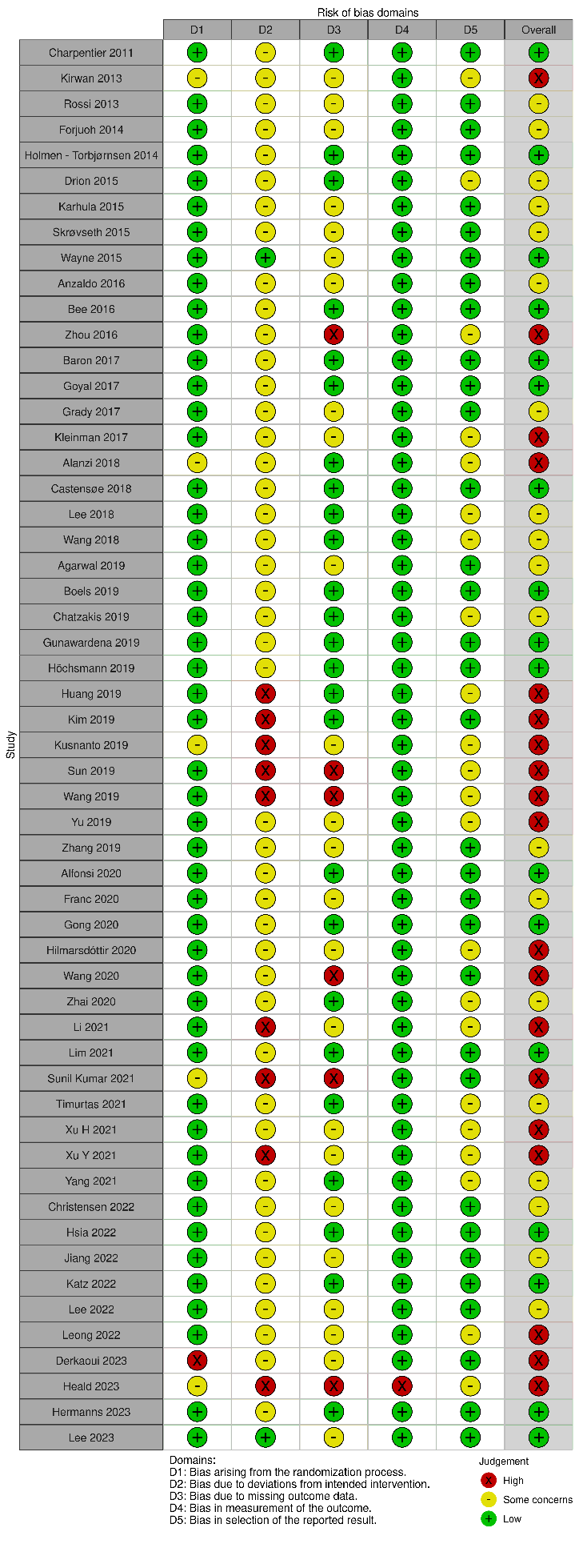
**

# **Supplementary Figure 9 – Study-level details of the risk of bias assessment for cluster-randomized studies**

**
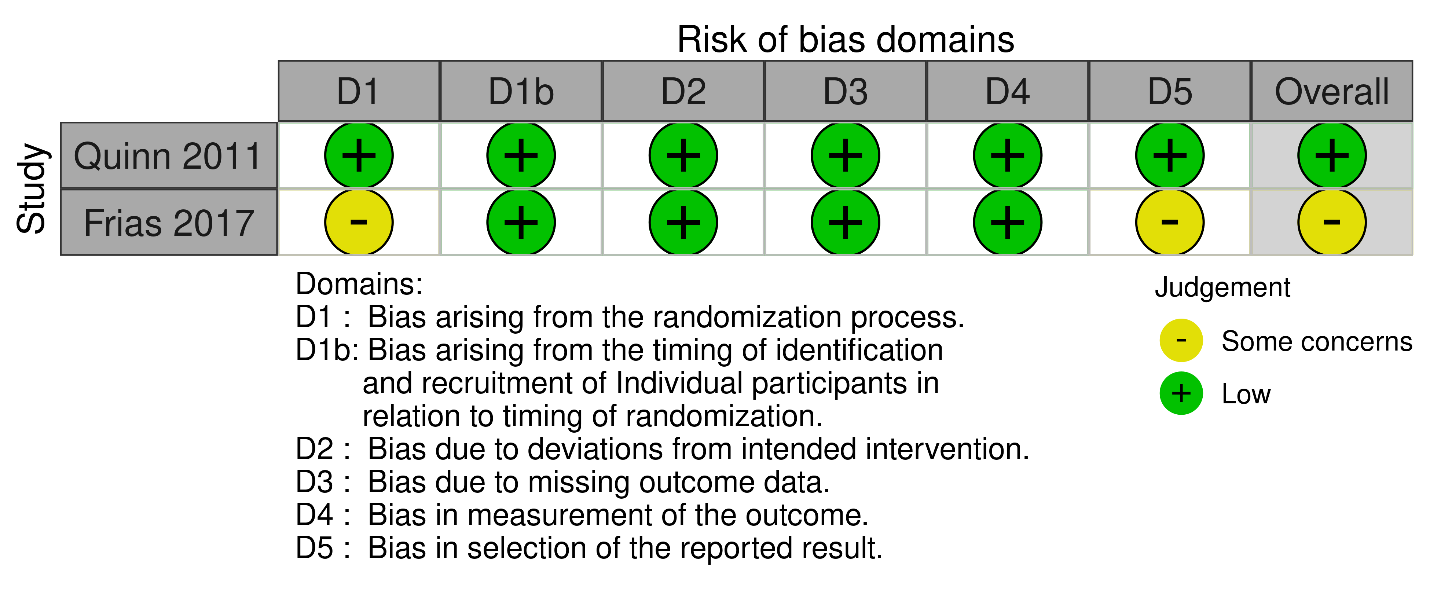
**

# **Supplementary Figure 10 – Meta-analysis of effect size at the 3-month time point (n=34 studies)**

**
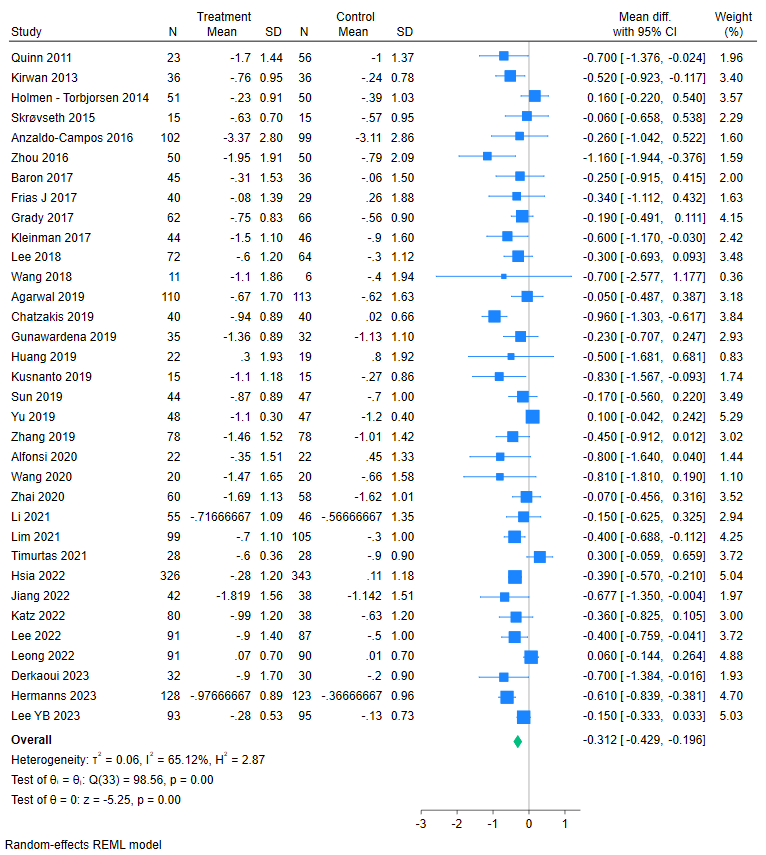
**

# **Supplementary Figure 11 – Meta-analysis of effect size at the 6-month time point (n=32 studies)**

**
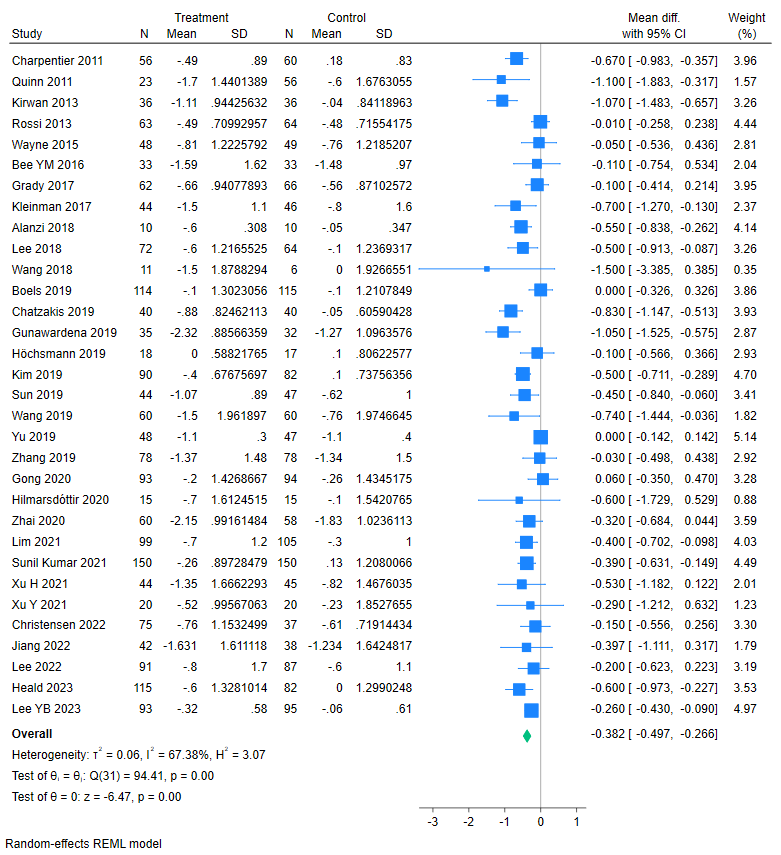
**

# **Supplementary Figure 12 – Meta-analysis of effect size at the 9-month time point (n=5 studies)**

**
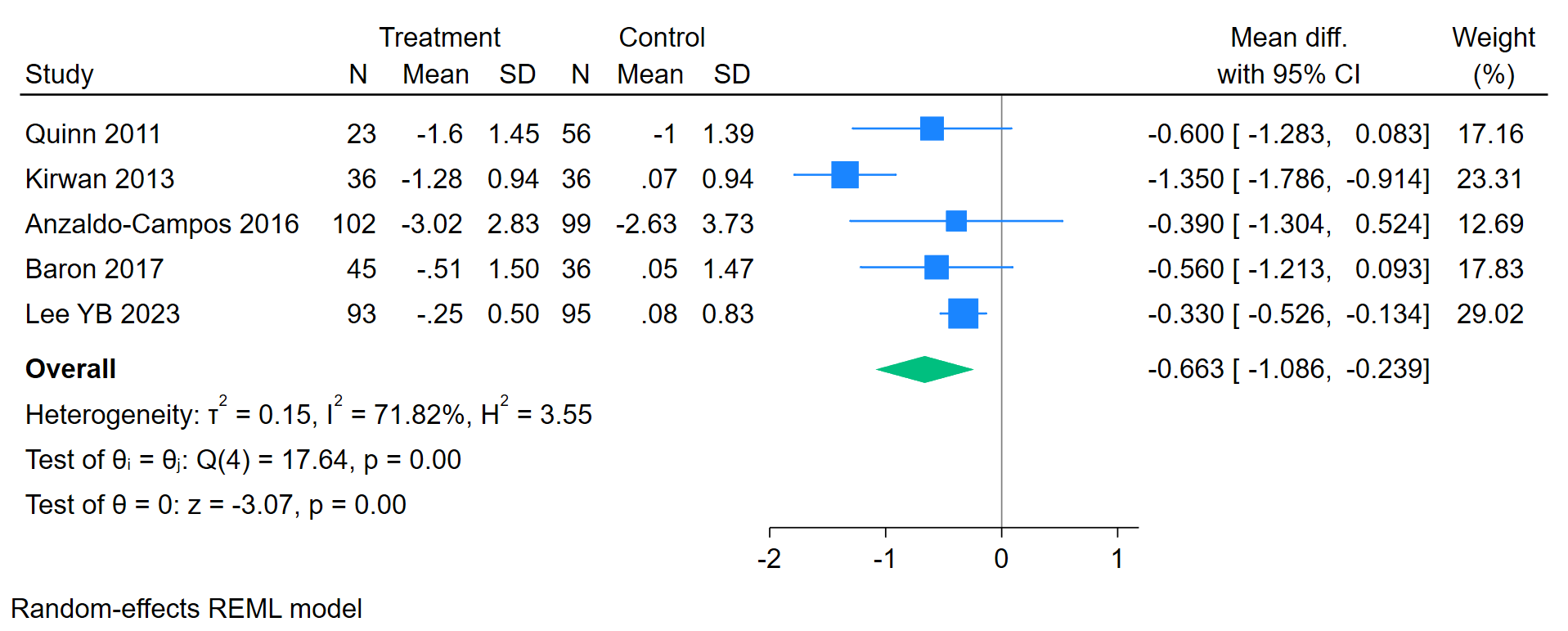
**

# **Supplementary Figure 13 – Meta-analysis of effect size at the 12-month time point (n=9 studies)**

**
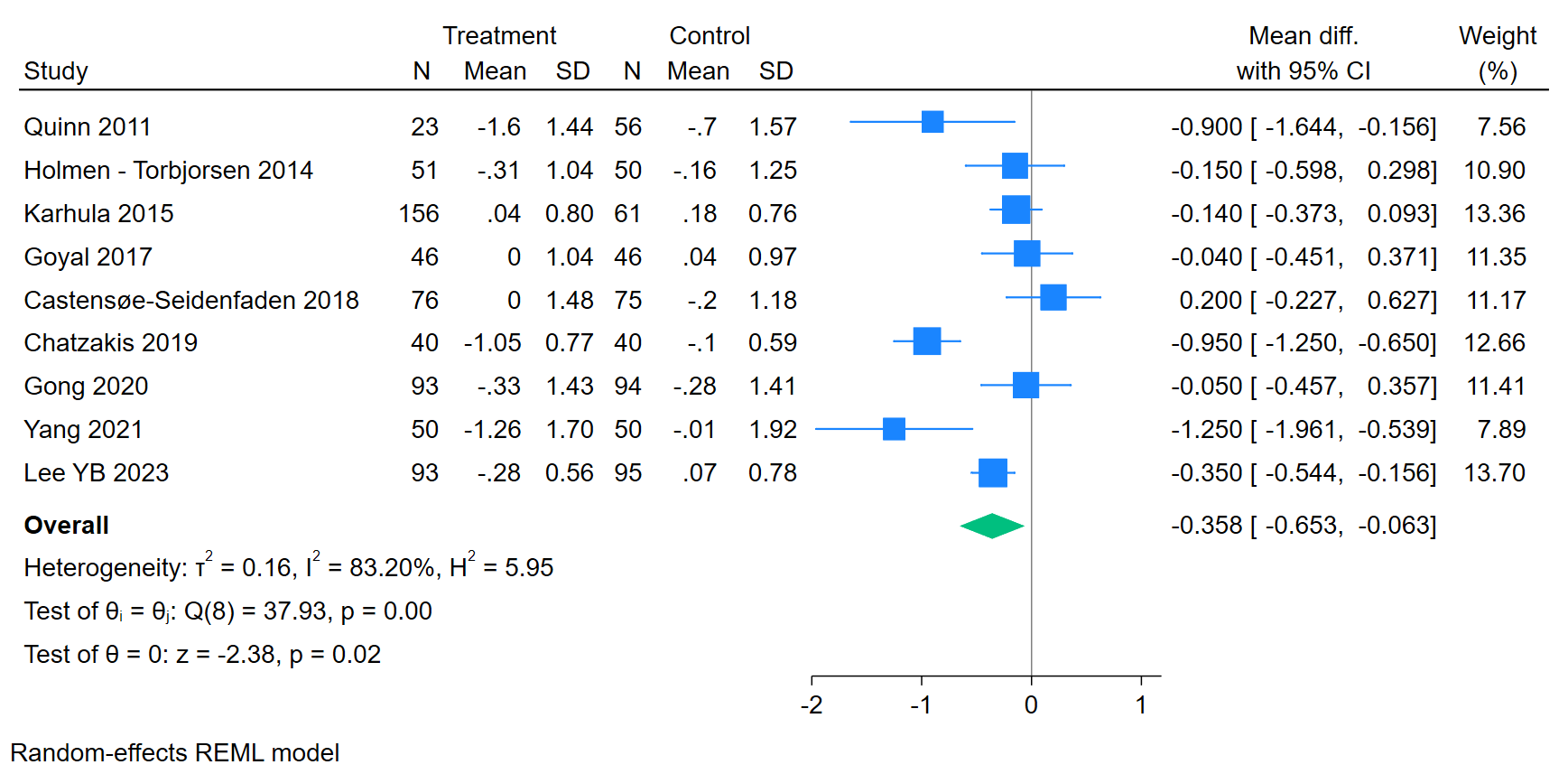
**

# **Supplementary Figure 14 – Contour-enhanced funnel plot to detect small-study effects**


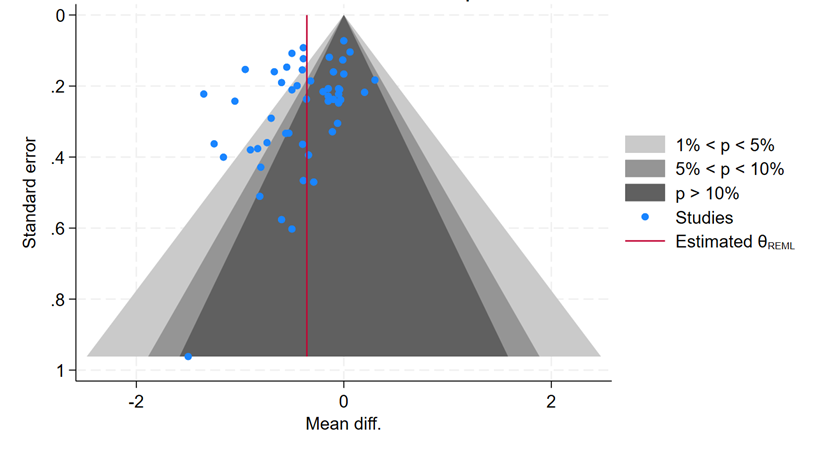

Supplement: Supplementary Data, Table and Figures [file mmc1.docx]
